# Genomics-driven discovery of a biosynthetic gene cluster required for the synthesis of BII-Rafflesfungin from the fungus *Phoma sp. F3723*

**DOI:** 10.1186/s12864-019-5762-6

**Published:** 2019-05-14

**Authors:** Swati Sinha, Choy-Eng Nge, Chung Yan Leong, Veronica Ng, Sharon Crasta, Mohammad Alfatah, Falicia Goh, Kia-Ngee Low, Huibin Zhang, Prakash Arumugam, Alexander Lezhava, Swaine L. Chen, Yoganathan Kanagasundaram, Siew Bee Ng, Frank Eisenhaber, Birgit Eisenhaber

**Affiliations:** 1 Bioinformatics Institute (BII), Agency for Science, Technology and Research (A*STAR), 30 Biopolis Street, #07-01 Matrix, Singapore, 138671 Republic of Singapore; 2 Genome Institue of Singapore (GIS), Agency for Science, Technology and Research (A*STAR), 60 Biopolis Street, #02-01 Genome, Singapore, 138672 Republic of Singapore; 3 Department of Medicine, Yong Loo Lin School of Medicine, National University of Singapore, 1E Kent Ridge Road, NUHS Tower Block, Level 10, Singapore, 119228 Republic of Singapore; 4 School of Computer Science and Engineering (SCSE), Nanyang Technological University (NTU), 50 Nanyang Drive, Singapore, 637553 Republic of Singapore

**Keywords:** Cyclic lipodepsipeptide, Biosynthetic gene cluster, Combined NRPS/PKS cluster, NonRibosomal Peptide Synthetase (NRPS), Adenylation domain, Condensation domain, *Phoma* species

## Abstract

**Background:**

Phomafungin is a recently reported broad spectrum antifungal compound but its biosynthetic pathway is unknown. We combed publicly available *Phoma* genomes but failed to find any putative biosynthetic gene cluster that could account for its biosynthesis.

**Results:**

Therefore, we sequenced the genome of one of our *Phoma* strains (F3723) previously identified as having antifungal activity in a high-throughput screen. We found a biosynthetic gene cluster that was predicted to synthesize a cyclic lipodepsipeptide that differs in the amino acid composition compared to Phomafungin. Antifungal activity guided isolation yielded a new compound, BII-Rafflesfungin, the structure of which was determined.

**Conclusions:**

We describe the NRPS-t1PKS cluster ‘*BIIRfg’* compatible with the synthesis of the cyclic lipodepsipeptide BII-Rafflesfungin [HMHDA-L-Ala-L-Glu-L-Asn-L-Ser-L-Ser-D-Ser-D-allo-Thr-Gly]. We report new Stachelhaus codes for Ala, Glu, Asn, Ser, Thr, and Gly. We propose a mechanism for BII-Rafflesfungin biosynthesis, which involves the formation of the lipid part by BIIRfg_PKS followed by activation and transfer of the lipid chain by a predicted AMP-ligase on to the first PCP domain of the BIIRfg_NRPS gene.

**Electronic supplementary material:**

The online version of this article (10.1186/s12864-019-5762-6) contains supplementary material, which is available to authorized users.

## Background

Despite the general reluctance and the slowly changing attitude of pharmaceutical and biotech industries to explore secondary metabolites of plants and microbes for pharmaceutical applications during the last two decades [1], more than a third of recently approved medicines are still natural products or have been derived from lead compounds found in living organisms [2–6]. In the field of antibacterial and antifungal compounds, inputs from natural product biology are particularly indispensable. Recent sequencing outputs from many microbes including bacteria and fungi support their potential role as a rich source pool for compounds with broad pharmacological relevance.

Nonribosomal peptides and polyketides represent a large class of natural products. Despite their immense structural and functional diversity, they are synthesized by strikingly similar multimodular enzymes called nonribosomal peptide synthetases (NRPSs) and polyketide synthases (PKSs), respectively [7–9]. The sequences of both types of enzymes, NRPSs and PKSs, consist of modules where each module is thought to be responsible for catalyzing the attachment of a specific substrate on-to the growing chain in an assembly-line like manner [7, 8]. Typically, amino acids (NRPS) or simple carboxylic acids (PKS) are the substrates added by one module. A module consists of essential (core) domains but it is possible that it harbors additional auxiliary domain(s). The immense structural diversity of nonribosomal peptides and polyketides can be achieved by varying the number and/or order of modules with different combinations of both core domains and auxiliary domains [7, 8].

In the case of NRPSs, a typical module has at least three core domains, an adenylation domain (A domain), a peptidyl carrier protein (PCP; also known as thiolation domain, i.e. T domain) and a condensation domain (C domain). The A domain selects and activates the cognate amino acid by adenylation [10, 11]. The activated amino acid adenylate is then transferred to a PCP, which transports the activated intermediate to a C domain [12]. The PCP domain carries a phosphopantetheinyl at a conserved serine (Ser) residue, which supports the transportation of substrates between the active sites of the domains. The C domain finally catalyzes the formation of the peptide bond between the thioester group of the elongating peptide chain from the earlier module with the amino group of the current module. It has been inferred that the enzyme has an acceptor site for the nucleophile and a donor site for the electrophile [13–16].

For PKSs, a typical module contains an acyl carrier protein (ACP), a ketosynthase (KS), an acyltransferase (AT) domain and a thioesterase (Te) domain, which all together catalyse the linear extension of a polyketide intermediate by two carbon atoms [17–19]. The building block of a polyketide chain is usually an acyl-CoA or a carboxyacyl-CoA which is loaded on the AT domain. The AT domain transfers the activated building block chain to the neighbouring ACP domain [20]. The ACP domain, like the PCP domain of NRPSs, is 4′-phosphopantetheinylated and transfers the intermediate to the active site (cysteine) of the upstream KS domain. The KS domain catalyses a Claisen-like condensation reaction by forming a carbon-carbon bond between the thioester carbonyl of the ACP-bound polyketide chain and the alpha carbon atom of the extending unit. This decarboxylative condensation step yields a ketide chain with a β-keto group [8, 21]. The β-keto group is optionally modified by additional auxiliary domains like ketoreductase (KS), dehydratase (DH), or enoyl reductase (ER) domains before the growing polyketide chain is released as a linear or cyclic product via the C-terminal thioesterase (Te; termination) domain [8, 17]. Of note, most of the fungal PKS belong to modular iterative type I-PKS, which is a single, giant protein consisting of only one module using the same set of domains of this module in an iterative manner to build the final polyketide [7, 21, 22].

**Fig. 1:**
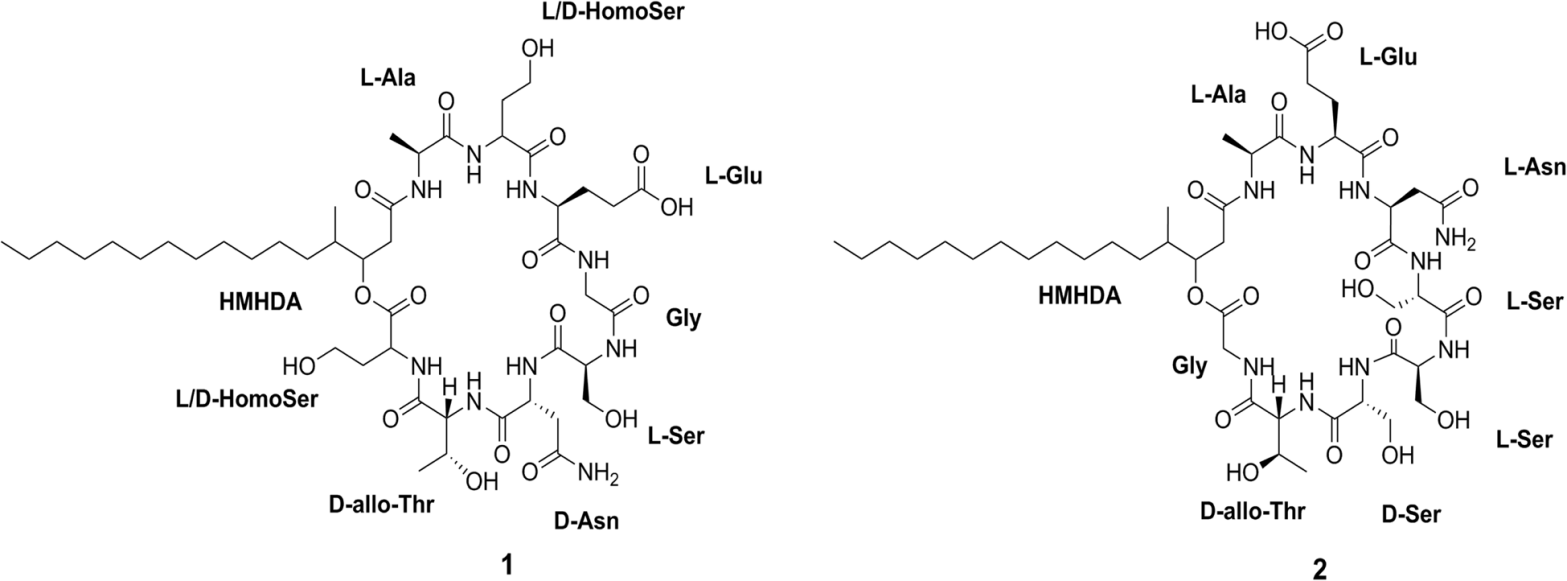
Structures of Phomafungin (**1**) and BII-Rafflesfungin (**2**)

Recently, cyclic lipodepsipeptides, a sub-class of nonribosomal peptides, have attracted a great deal of attention for discovery of new antibiotics [23, 24]. These peptides contain one or more ester bonds in addition to amide bonds. Phomafungin (**1**) is a known cyclic lipodepsipeptide which is produced by a widespread tropical *Phoma* sp. It was initially identified, isolated and characterized based on a *Candida albicans* fitness test profile (CaFT) [25]. The compound was shown to have broad-spectrum antifungal activity against human fungal pathogens including *Candida* sp*.* Its structure resembles other cyclic lipodepsipeptides like Syringomycin E [26], Pseudomycin A [27], Cormycin A [28], and Phaeofungin [29]. It consists of a closed ring of eight amino acids (Homoserine-Threonine-Asparagine-Serine-Glycine-Glutamate-Homoserine-Alanine) linked to a fatty acid lipid hydrocarbon tail of β-hydroxy-γ-methyl-hexadecanoic acid (HMHDA) as shown in Fig. 1. Until recently, there has been no further research on this compound and there is no reported biosynthetic gene cluster for its synthesis.

In this study, we report the discovery of a new cyclic lipodepsipeptide variant, BII-Rafflesfungin, from a marine *Phoma* species. It structurally resembles Phomafungin but differs in the amino acid composition of the closed ring structural subunit; it also retains the expected antifungal activity. Analysis of the genome sequence identified a combined NRPS-t1PKS biosynthetic gene cluster consistent with a role in the biosynthesis of this new compound.

## Results

### Sequencing and sequence annotation of the genome from *Phoma* strain F3723

The discovery of Phomafungin with distinct antifungal activity [25] without any identification of its biosynthetic genes or enzymes motivated us to analyse the publicly available *Phoma* genomes: *Phoma herbarum* (GCA_001599375.1) and, more recently, *Phoma koolunga* (GCA_004151575.1, GCA_004151145.1, GCA_004151165.1). Unfortunately, no NRPS/PKS was detected that could be speculated as the correct gene cluster for the synthesis of Phomafungin (for details see Additional File 1: Table S1). Because we did not have access to the original *Phoma* strain from which Phomafungin was isolated, we tested extracts derived from *Phoma* strains in a large strain collection [5] for antifungal activity and found that the extract of one strain, F3723, had growth inhibitory activity against *Candida albicans* (data not shown).

We used PacBio SMRT sequencing to determine the genome sequence of *Phoma* strain F3723. The 18S rRNA of F3723 is 99.8% identical with 18S rRNA of *Phoma MJ76 (*E-value = 0.0*,* Accession: HM590661) confirming the original species annotation in the library [5]. This finding is further supported by the phylogenetic analysis based on the standard protein coding marker gene, beta-tubulin, where F3723 clusters together with all other *Phoma* species (Additional File 2: Figure S1).

**Fig. 2:**
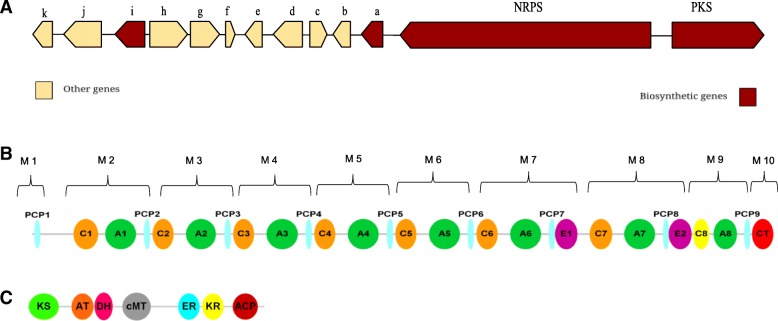
The predicted biosynthetic gene cluster *BIIRfg*. **a** Gene organization of the predicted biosynthetic gene cluster. Red arrows show the biosynthetic genes while yellow ones are other genes present in the vicinity. The direction of arrows corresponds to that of the reading frame. The neighboring orfs of NRPS and PKS genes are labelled a, b, c, d, e, f, g, h, i, j and k. **b** Domain structure of the NRPS gene: The green ovals labelled An (*n* = 1..8) represent adenylation domains. The orange ovals labelled Cn (*n* = 1..8) represent condensation domains. The predicted condensation domain, which was not predicted by antiSMASH, is shown in yellow oval. The last condensation domain is labelled as CT and is shown in red oval. The pink ovals labelled En (*n* = 1,2) represent epimerization domains. Cyan ovals labelled PCPn (*n* = 1..9) represent peptidyl carrier protein (PCP) domains. The constituent modules in the cluster are marked from 1 to 10. **c** Domain structure of PKS gene: T1-PKS module consists of a beta-ketoacyl synthase (KS) domain shown in green, acyltransferase (AT) domain in orange, dehydratase (DH) domain in magenta, methyltransferase (cMT) domain in grey, enoyl reductase (ER) domain in cyan, ketoreductase (KR) in yellow and acyl carrier protein (ACP) domain in red

The resulting sequences including their protein-coding stretches, predicted by using AUGUSTUS (version 3.2.2) [30] (11,055 predicted protein sequences) and GeneMark-ES (version4.10) [31, 32] (11,687 predicted protein sequences), were analysed both with our in-house ANNOTATOR pipeline [33] as well as with antiSMASH (Version 3.0.3) [34] to detect any biosynthetic gene clusters consistent with the production of Phomafungin. There was no exact match but we found a single almost complete gene cluster having one NRPS gene with eight A domains which is consistent with the presence of eight amino acid residues in Phomafungin. Next to this cluster, as shown in Fig. 2a, we found genes encoding a type I-PKS module which could possibly synthesize the lipid part of Phomafungin. Of note, a type II thioesterase domain containing protein (orf-a) and an AMP-dependent ligase (orf-i) were also found in the vicinity of the NRPS/PKS genes.

### Detailed analysis of the NRPS gene architecture

According to the antiSMASH output, the NRPS gene in the cluster consists of 27 domains organized in 10 modules (M); M1 = PCP1, M2 = C1-A1-PCP2, M3 = C2-A2-PCP3, M4 = C3-A3-PCP4, M5 = C4-A4-PCP5, M6 = C5-A5-PCP6, M7 = C6-A6-PCP7-E1, M8 = C7-A7-PCP8-E2, M9 = A8-PCP9, M10 = C_T_. The initiation module, M1, consists of a single PCP domain which is sufficient to accept the fatty acid intermediate for further elongation of the peptide chain by subsequent downstream domains [35–38]. Each of the modules M2-M9 is thought to be responsible for catalyzing the attachment of one amino acid to the growing chain of the compound, in total eight amino acids. In accordance with their functionality, modules M2-M9 are expected to be repetitive blocks of C-A-PCP domains. We observed that modules M2-M8 were complete but module 9 (M9) lacked the C domain. Modules 7 and 8 (M7 and M8) harbor additional E domains M7 = C6-A6-PCP7-**E1** and M8 = C7-A7-PCP8-**E2** besides the typical C-A-PCP domains. The termination module M10 consists of the terminal condensation domain C_T_ which is believed to help in cyclization of the peptide as reported previously in different studies [37, 38]. Unlike bacterial NRPS systems, where the terminating domain is usually a thioesterase domain, fungi typically have this type of specialized C_T_ domain [39–41].

**Fig. 3:**
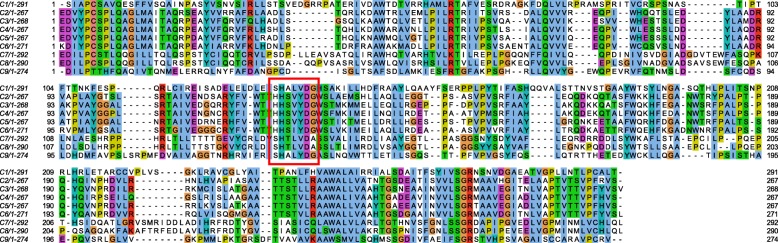
Alignment of all nine condensation domains of the NRPS gene cluster. The alignment also includes the C8 condensation domain which was not predicted by antiSMASH. The conserved motif HHxxxDG is shown in red box. For C1, C7, C8 and C9 (the last condensation domain which acts as termination domain) the first H (His) is replaced by S (Ser) but the functionally important second H (His) residue of the motif is found conserved in all the condensation domain sequences

We were able to identify the missing C domain in module 9 based on a HMMER search [42] within our ANNOTATOR [33] environment with a profile created from the other eight C domains of the cluster against the translated protein sequence of the cluster region between E2 (end of module M8) and A8 (module M9). We got a single significant hit (E-value = 8.5e-82, see details in Additional File 3: Figure S2). Figure 3 shows an alignment of all nine C-domains from the cluster. Of note, all known functionally important residues were conserved. This suggests that the NRPS gene encodes a complete enzyme that could synthesize a depsipeptide consisting of eight amino acids. Its predicted complete structure (28 domains, 10 modules) is shown in Fig. 2b.

### Identification of specificity conferring codes for the adenylation domains reveals that the NRPS cluster is not compatible with the biosynthesis of Phomafungin

Deep sequence analysis of all A domains of the NRPS gene was done to identify the specificity determining codes of the A domains [43, 44]. For this purpose, all A-domains from the NRPS gene were aligned, using MAFFT [45], with the phenylalanine activating adenylation domain (PheA) of gramicidin S synthetase (GrsA; P0C061 as reference sequence [11, 46–48]. This alignment was used to predict amino acid residues responsible for substrate specificity. Table 1 summarizes these amino acids also known as Stachelhaus codes (according to the GrsA Phe numbering) [11] of the NRPS A domains, A1 to A8. To our great surprise, domains A4, A5 and A6 display the identical Stachelhaus code **‘DVQTVMAI’** and, therefore, are expected to activate the same amino acid. However, Phomafungin does not contain three identical amino acid residues. Thus, the NRPS cluster described here is not compatible with the biosynthesis of the peptide part of Phomafungin. Consequently, we hypothesized that F3723 may synthesize a novel variant of Phomafungin.

**Table 1:** Stachelhaus codes for the adenylation domains (A1-A8) of the NRPS gene cluster. The residues are mapped based on positions according to GrsA-Phe numbering. The predicted activated AAs are listed in column 3

Protein	Module	Activated AA	Residue positions according to GrsA Phe numbering							
			235	236	239	278	299	301	322	330
A_1_	M2	**Ala**	D	V	A	T	I	T	A	I
A_2_	M3	**Glu**	D	V	T	H	S	G	S	V
A_3_	M4	**Asn**	D	V	S	N	V	G	S	I
A_4_	M5	**Ser**	D	V	Q	T	V	M	A	I
A_5_	M6	**Ser**	D	V	Q	T	V	M	A	I
A_6_	M7	**Ser**	D	V	Q	T	V	M	A	I
A_7_	M8	**Thr**	D	A	Q	T	I	M	A	I
A_8_	M9	**Gly**	D	V	S	N	V	I	G	I

### Towards the structure of the new compound BII-Rafflesfungin (2)

Since the extract derived from F3723 was previously shown to possess antifungal activity against *Candida albicans*, we used a *C. albicans* whole-cell assay (see Methods, “Antifungal assay”) to guide purification of the bioactive compound. This yielded a light yellowish oil, which we denote as BII-Rafflesfungin, with an ESIMS [M + H]^+^ peak at *m/z* 1002 and a molecular formula of C_44_H_75_N_9_O_17_ based on HRESIMS measurement. Notably, the mass of the new compound is 28 units smaller than that reported for Phomafungin, which would be consistent with the absence of two -CH_2_- groups [25].

The ^13^C NMR showed a total of 44 carbon resonances, comprising 19 methylenes (5 overlapped), 10 methines, 4 methyls, and 11 carbonyl carbon atoms. The observed 11 carbonyl signals resonating at δ 168.8–174.4 in the ^13^C NMR, together with the observed large methylene signals observed at δ 1.22–1.31 in the ^1^H NMR, suggested a lipopeptide moiety (Table 2). Further analysis of the HSQC, HMBC, and COSY spectra (see Methods section and Additional file 4: Figures. S3-S7) revealed the presence of 8 amino acid (an alanine (Ala), a glutamic acid (Glu), an asparagine (Asn), three serines (Ser), a threonine/allo-threonine (Thr/allo-Thr), and a glycine (Gly)) moieties, incorporating a fatty acyl (FA) group resembling the arrangement in Phomafungin [25]. The observed 28 mass units (corresponding to two -CH_2_- groups) lesser in BII-Rafflesfungin compared to Phomafungin are consistent with the replacement of two homoserine (Homoser) units in Phomafungin by two serine (Ser) units in BII-Rafflesfungin. The fatty acyl group in BII-Rafflesfungin was identified as β-hydroxy-γ-methyl hexadecanoic acid (HMHDA) based on the 1D, 2D NMR, and HRESIMS data being identical to those of Phomafungin [25].

**Table 2:** ^1^H (400 MHz) and ^13^C (100 MHz) NMR data of 2 in DMSO-*d*_6_^*a*^

AA	Position	Mult	δ_C_	δ_H_ (*J* in Hz)
L-Ala	1	CO	172.6	–
	2	CH	48.4	4.22 m
	3	CH_3_	17.8	1.20 d (7.1)
		NH	–	7.89 br d (6.9)
L-Glu	1	CO	171.3	–
	2	CH	52.5	4.19 m
	3	CH_2_	26.7	1.75 m, 1.93 m
	4	CH_2_	30.3	2.22 m, 2.22 m
	5	COOH	174.4	–
		NH	–	8.18 d (7.2)
L-Asn	1	CO	171.1	–
	2	CH	49.6	4.50 dt (7.4, 5.6)
	3	CH_2_	36.8	2.65 dd (15.5, 5.6), 2.73dd (15.5, 5.6)
	4	*C*ONH_2_	172.3	–
		NH	–	8.12 br d (7.4)
		CO*NH*_*2*_	–	7.03 br s, 7.54br s
L-Ser	1	CO	170.2^*a*^	
	2	CH	57.0	4.11 q (5.4)
	3	CH_2_	60.9	3.68 m, 3.68 m
		NH	–	8.15 br s
L-Ser	1	CO	170.1^*a*^	
	2	CH	56.3	4.27 m
	3	CH_2_	61.2	3.64 m, 3.64 m
		NH	–	8.21 br s
L-Ser	1	CO	170.1^*a*^	–
	2	CH	55.1	4.36 dt (7.8, 5.6)
	3	CH_2_	61.8	3.65 m, 3.65 m
		NH	–	7.75br d (7.8)
D-allo-Thr	1	CO	170.0^*a*^	–
	2	CH	58.4	4.25 m
	3	CH	66.5	3.93 m
	4	CH_3_	19.6	1.07 d (6.3)
		NH	–	7.93br d (8.8)
Gly	1	CO	168.8	–
	2	CH_2_	40.9	3.78dd (17.3, 5.5), 3.87dd (17.3, 5.5)
		NH	–	7.96br t (5.5)
HMHDA	1	CO	169.4	–
	2	CH_2_	36.9	2.32 m, 2.35 m
	3	CH	75.4	5.04 m
	4	CH	35.8	1.71 m
	5	CH_2_	31.7	1.03 m, 1.33 m
	6	CH_2_	26.4	1.22 m, 1.31 m
	7–13	CH_2_	28.7–29.2	1.22 m–1.28 m
	14	CH_2_	31.3	1.24 m, 1.24 m
	15	CH_2_	22.1	1.24 m, 1.29 m
	16	CH3	13.9	0.86 t (6.6)
	17	CH_3_	14.6	0.83 d (6.6)

^*a*^Assignments based on COSY, HSQCED and HMBC. ^1^H (400 MHz) and ^13^C NMR (100 MHz) data were referenced to DMSO-*d*_6_ (δ_Η_ 2.49 and δ_C_ 39.50). Chemical shifts (δ) in ppm. s: singlet; d: doublet. q: quartet; m: multiplet; br: broad; Chemical shifts (δ) in ppm. ^b^Assignments are interchangeable

**Fig. 4:**
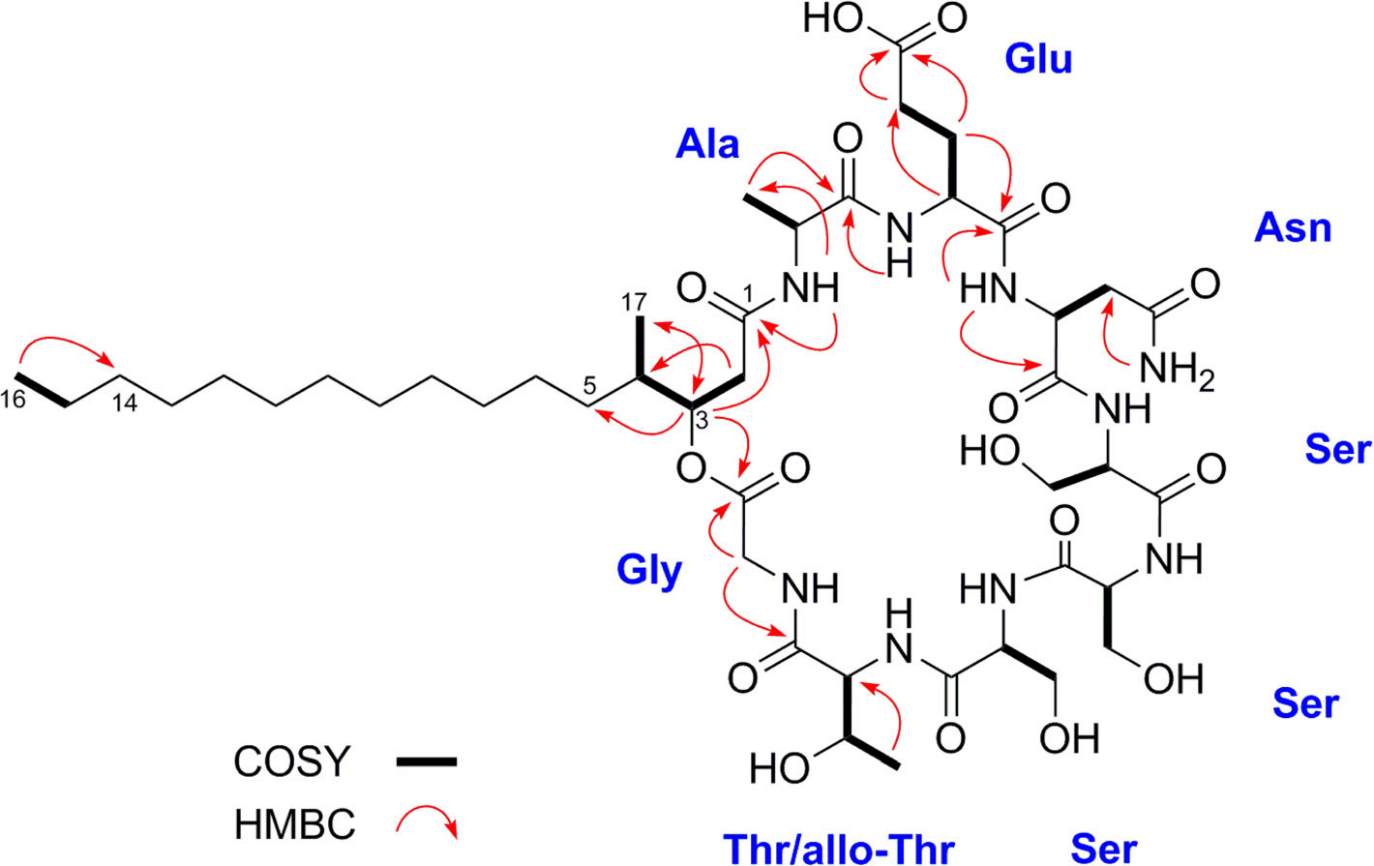
Cosy and HMBC correlations of BII-Rafflesfungin. The sequential positions of the three serines and of threonine could not be determined based on these methods

**Fig. 5:**
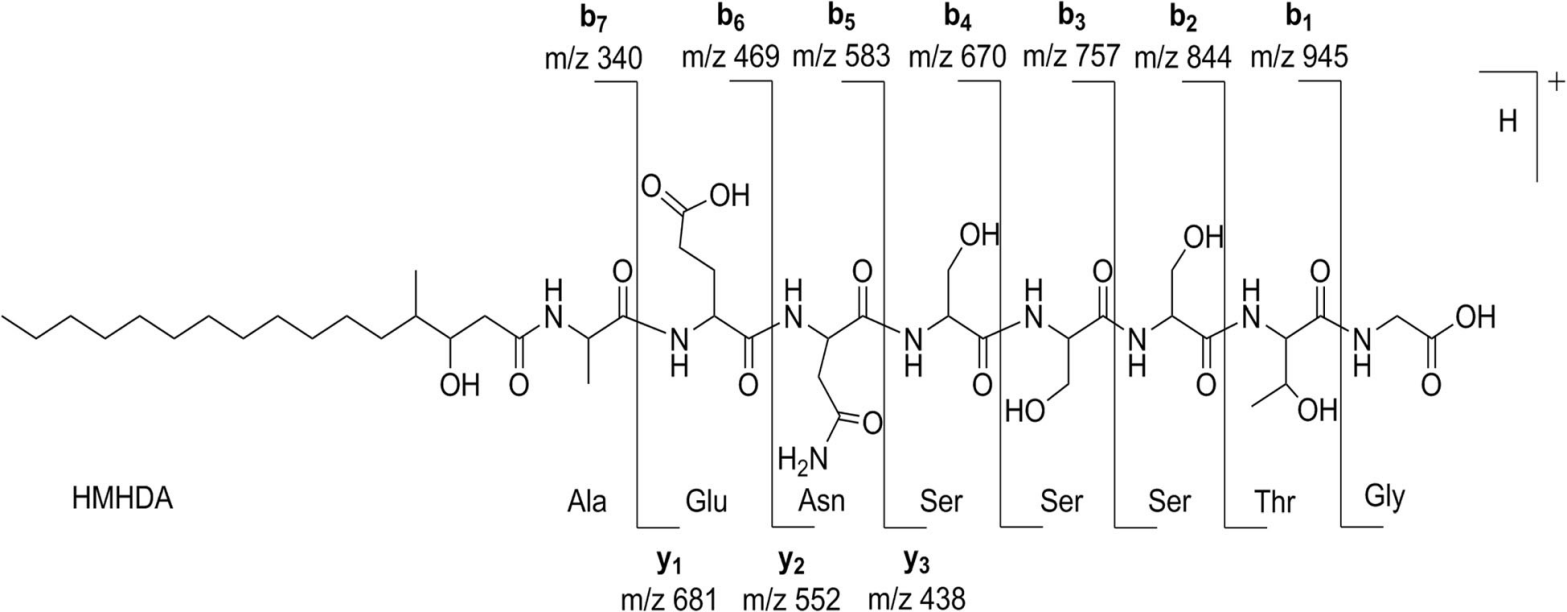
MS2 fragments of the hydrolysed product (linear peptide)

We were able to establish a partial sequence of BII-Rafflesfungin as Gly-HMHDA-Ala-Glu-Asn through the HMBC couplings from α-protons and/or α-NH to carbonyl carbons of adjacent residues (Fig. 4). But, based on this method, the exact positions of the three serine and the threonine/allo-threonine residues in the peptide could not be determined. The planar structure of BII-Rafflesfungin was subsequently confirmed by HRESIMS/MS analysis of the hydrolysed product (linear peptide) which gave a pseudo molecular ion [M + H]^+^ at *m/z* 1020. The HR-MS/MS analysis of this linear peptide produced both b- and y-type fragments and is shown in Fig. 5. The b series fragment ions observed at *m/z* 945, 844, 757, 670, 583, 469, and 340 indicate sequential losses of Gly, Thr, Ser, Ser, Ser, Asn, Glu. Therefore, we establish the sequence of BII-Rafflesfungin as HMHDA-Ala-Glu-Asn-Ser-Ser-Ser-Thr-Gly with predicted structure as shown in **(2)** in Fig. 1. To note, the predicted structure of BII-Raflesfungin is in full agreement with the partial findings from the sequence analysis of the NRPS cluster.

To conclude, there is experimentally verified evidence that the *Phoma* species reported in the present study produces a new cyclic lipodepsipeptide that we named *BII-Rafflesfungin*. Its planar structure is as shown in (**2)** in Fig. 1. The related biosynthetic gene cluster was named as *‘BIIRfg’* and the NRPS gene responsible for the biosynthesis of the cyclic peptide core of BII-Rafflesfungin was named as *‘BIIRfg_NRPS’*. The sequence of *BIIRfg* was submitted to Genbank (Accession number MK043052).

### Revisiting the Stachelhaus codes of the *BIIRfg* NRPS adenylation domains: discovery of 6 new Stachelhaus codes

Even without knowing the exact sequence of BII-Rafflesfungin, we were able to predict some new Stachelhaus codes [9] (see Table 1) from the amino acid composition and the NRPS cluster sequence for BII-Rafflesfungin. The identical signatures for A4, A5, and A6 should code for serine as this is the only amino acid with three occurrences in the sequence. Further, the signature code for the A7 domain is ‘DAQTIMAI’ which is very similar to the code ‘DVQTVMAI’ for the serine-activating domain. Given that serine and threonine are structurally similar with polar uncharged side chains, we predicted that the A7 domain is a threonine-activating domain. In addition, we were able to predict A2 as a glutamic acid-activating domain based on similarity with the known codes [41]. Also, the NRPSPredictor2 [44] suggests the most probable Stachelhaus code to be Glu.

Based on the experimental structural elucidation of the new compound BII-Rafflesfungin, we were able to fill in all the gaps in Table 1 regarding the information about which adenylation domain selects/activates which amino acid. The predicted Stachelhaus codes of the adenylation domains A2 and A7 are in good agreement with the experimental findings. Most importantly, the three identical Stachelhaus codes for adenylation domains A4, A5 and A6 code for the same amino acid serine.

To our best knowledge, none of the new Stachelhaus codes found in our analysis for 6 amino acids (Ala, Glu, Asn, Ser, Thr, Gly; Table 1) was described before. Our result is fully supported by theoretical considerations and experimental findings.

### Attempts towards the stereochemistry: sequence analysis of functional subtypes of condensation domains

In order to identify the subtypes of the eight condensation domains and to predict the stereochemistry of the amino acids, we carried out HMMER searches against the HMM models of ^D^C_L_, ^L^C_L_, starter and dual type of C domains. The HMM models were created from the alignments provided by Rausch et al. [49]. Unfortunately, the result of this analysis was inconclusive because all the C domains were highly scored by the ^D^C_L_ HMM model (Additional file 5: Table S2). This result suggests that all amino acids seem to be D amino acids. However, there is a contradictory line of evidence. For further investigation, we performed a phylogenetic tree analysis of the functional distribution of all the C domains using MEGA7 [50] (Maximum Likelihood (ML) tree). Surprisingly, all the C domains of the NRPS gene were found bundled with dual type C domains despite of being highly scored by the ^D^C_L_ type HMMER models (Additional file 6: Figure S8).

Of note, the NRPS gene harbors two epimerization domains (M7, Ser; M8, Thr) which typically epimerize the amino acid of the growing peptide chain into D configuration. Both domains seem to be functionally intact: We find the functionally important motif/active site HHxxxDxVSW [51] conserved in both sequences (Additional file 7: Figure S9). Interestingly, a HMMER search with the sequences of both epimerization domains E1(M7) and E2 (M8) against enzyme classification (EC) profiles [52, 53] resulted in significant hits to aspartate racemaces (EC 5.1.1.13) with E-values of 6.1 e-17 and 4.8 e-15, respectively. Therefore, we speculate that Ser7 and Thr 8 undergo stereoisomerization. But, to conclude, we cannot deduce the exact functional subtypes of the C domains of the NRPS gene and the stereochemistry of the amino acids from sequence-analytic considerations. This issue requires additional experimental clarification that is described below.

### Experimental determination of the stereochemistry of BII-Rafflesfungin

In order to determine the absolute configuration of the amino acid residues, BII-Rafflesfungin (**2**) was hydrolyzed with 6 N HCl and the acid hydrolysate was derivatized with Nα-(2,4-dinitro-5-fluorophenyl)-L- alaninamide (L-FDAA, Marfey’s reagent). The LCMS analysis of L-FDAA of (**2**) in comparison with the corresponding derivatives of D- and L-amino acid standards (Marfey’s method [54], see also Methods “Acid hydrolysis and derivatization of BII-Rafflesfungin”) indicated the exclusive presence of L-Ala, D/L-Ser, D-allo-Thr, Gly, L-Asn and L-Glu (Additional file 8: Figure S10). In the case of Ser, both the D- and the L-forms were identified. The observed ratio of L-serine: D-serine was 2:1 in the extracted ion chromatogram (EIC) suggesting the presence of two L-serine and one D-serine amino acids (Additional file 8: Figure S10). Of note, the FDAA-Asn residue was determined by comparison with the retention time of FDAA-Asp instead of FDAA-Asn due to the conversion of asparagine to aspartic acid during acid hydrolysis. We did not determine the absolute configuration of HMHDA.

There is full agreement between the architecture of the BIIRfg_NRPS gene (Fig. 2b) and the experimental findings for the stereochemistry of BII-Rafflesfungin. The NRPS cluster contains three modules (M5, M6, M7) for catalyzing the attachment of the amino acid serine to the growing chain of the compound but only one of them (M8) harbors an epimerization (E) domain. This is a perfect match with the experimental finding that L-serine and D-serine were found in a ratio 2:1. The only other module that contains an epimerization domain is module M8 and this explains the finding of allo-Thr in the D configuration. All the other amino acid residues are found to be in the L configuration. To conclude, based on the combination of the experimental stereochemistry data and the analysis of the NRPS gene architecture, a cyclo-[HMHDA-L-Ala-L-Glu-L-Asn-L-Ser-L-Ser-D-Ser-D-allo-Thr-Gly] lipodepsipeptide structure (**2**) was established for BII-Rafflesfungin.

### The *BIIRfg* gene cluster contains a single type I polyketide synthase (t1-PKS), an AMP-dependent ligase, and a type II thioesterase domain containing protein in addition to the *BIIRfg* NRPS gene

Blastp searches and in-depth sequence analysis using ANNOTATOR [33] with the deduced coding sequences of the *BIIRfg* cluster were performed to find the gene functions encoded by neighboring ORFs of the *BIIRfg* NRPS gene. The best hits for each of the ORFs are shown in Table 3. There are three important findings that are possibly related to the biosynthesis of BII-Rafflesfungin: (i) There is a single type I polyketide synthase (t1-PKS) module localized next to the *BIIRfg* NRPS cluster (Fig. 2c). This module shows the presence of a beta-ketoacyl synthase (KS), acyltransferase (AT), dehydratase (DH), methyltransferase (cMT), enoyl reductase (ER) and ketoreductase (KR) domains. The initial observation does not show the presence of an acyl carrier protein (ACP) domain which is required to complete the PKS module. However, a blastn search of the nucleotide sequence of the predicted PKS region against the NCBI NT database resulted in a full-length hit to a putative polyketide synthase (Fpo_905) from *Fusarium poae* strain NRRL 26941 (E-value = 0.0). The domain organization of Fpo_905 is KS-AT-DH-cMT-ER-KR-ACP. A blastp search using the protein sequence of the ACP domain from Fpo_905 alone against the *BIIRfg_PKS* sequence resulted in a significant hit to a 57 amino acids long sequence located at the C-terminus of the PKS (E-value = 3e-29, 84% identity). A HMMER3 search on the same region against Pfam-30 database finds the PP-binding domain (Phosphopantetheine attachment site, PF00550) with an E-value of 4.1e-11. Therefore, we conclude, that the PKS gene cluster (named as *BIIRfg_PKS*) is complete and has a gene organization as shown in Fig. 2c. Notably, the *BIIRfg_PKS* cluster harbors a SAM-dependent methyltransferase. A HMMER3 search with its sequence shows that it belongs to class I methyltransferases (Methyltransf_23, PF13489, E-value = 3.3e-16). We find all functionally important residues [55] conserved (data not shown). This type of enzyme is compatible with the production of the lipid part of BII-Rafflesfungin, where there is a methyl group attached to C_γ_. (ii) Based on homology searches, orf-i is predicted to be an AMP-dependent ligase. A blastp search with orf-i against the NCBI non-redundant database finds VlmC, the reported AMP-dependent ligase from the verlamelin biosynthetic cluster (E-value = 0.0), and EcdI, the AMP-dependent ligase from the echinocandin biosynthesis cluster (E-value = 2e-122). The predicted AMP-ligase orf-i (573 AA) shows the presence of an AMP-binding domain (PF00501, E-value = 8.2e-45) and an AMP-binding enzyme C-terminal domain (PF13193, Evalue = 2.2e-15) similar to VlmC (580 AA) and EcdI (559 AA). An alignment of the predicted AMP-ligase orf-i along with EcdI and VlmC is provided in Additional file 9: Figure S11. (iii) orf-a (250AA) harbors a type II thioesterase domain (PF00975, E-value = 4.2e-7) which belongs to the alpha-beta hydrolase clan (CL0028). This finding is further supported by structural HHPred hits to 2K2Q_B (Surfactin synthetase thioesterase subunit: SrfAD) with an E-value of 4.8e-24 and to 3FLA_A (Rifamycin:RifR alpha-beta hydrolase thioesterase) with an E-value of 2.7e-17. The catalytic active site residues reported for SrfAD and RifR were also found to be conserved in orf-a. An alignment of the predicted TEII from BII-Rafflesfungin cluster (orf-a) along with SrfAD and RifR is shown in Additional file 10: Figure S12. The alignment highlights the conserved active site triad using red triangles in all the three sequences, S82-D192-H217 in orf-a; S94-D200-H228 in RifR [56], and **S86-**D189-H216 in SrfAD [57].

**Table 3:** Genes located in and adjacent to the *BIIRfg* biosynthetic gene cluster. The predicted function of the genes is based on the in-house sequence annotation pipeline ANNOTATOR. The best blast hit is reported. The observed E-value and percent identity for each gene are shown in parentheses. The rows in bold show the genes thought to be involved in the biosynthesis of BII-Rafflesfungin

Gene (protein_id)	Predicted Function	Best blast hit (E-value, % Identity)
BIIRfg_NRPS (QCC62999.1)	**Nonribosomal peptide synthase**	**GAM84991.1** **[** ***Fungal sp. 11,243*** **]** **(0.0, 43%)**
BIIRfg_PKS (QCC63000.1)	**Polyketide synthase**	**ALQ32757.1** **[** ***Fusarium aywerte*** **]** **(0.0, 70%)**
orf-a (QCC62998.1)	**Thioesterase type II (PF00975)**	**KPA42378.1** **[** ***Fusarium langsethiae*** **]** **(1e-117, 66%)**
orf-b (QCC62997.1)	Hypothetical RTA1 (PF04479) domain containing protein	OQO12260.1 [*Rachicladosporium antarcticum*] (3e-133, 60%)
orf-c (QCC62996.1)	Hypothetical protein	OBS28051.1 [*Fusarium poae*] (2e-67, 51%)
orf-d (QCC62995.1)	FAD binding domain (PF01565) containing protein	OWY43811.1 [*Alternaria alternata*] (0.0, 79%)
orf-e (QCC62994.1)	Similar to heme oxygenase	KPA35830.1 [*Fusarium langsethiae*] (2e-85, 63%)
orf-f (QCC62993.1)	MARVEL domain (PF01284) containing protein	EEH09252.1 [*Histoplasma capsulatum*] (3e-58, 56%)
orf-g (QCC62992.1)	Putative membrane transport protein (Major facilitator superfamily, PF07690)	KXL45980.1 [*Acidomyces richmondensis*] (0.0, 54%)
orf-h (QCC62991.1)	Glycosyltransferase (PF03033)	EEH09251.1 [*Histoplasma capsulatum*] (0.0, 70%)
orf-i (QCC62990.1)	**AMP dependent ligase (PF00501)**	**KPA42376.1** **[** ***Fusarium lansethiae*** **]** **(0.0,69%)**
orf-j (QCC62989.1)	Hypothetical P-loop containing ABC transporter protein	OKO93750.1 [*Penicillium subrubescens*] (0.0, 34%)
orf-k (QCC62988.1)	Heme binding (PF00141)	KZM26722.1 [*Ascochyta rabiei*] (0.0, 89%)

To conclude, with a single type I polyketide synthase (t1-PKS), an AMP-dependent ligase, a type II thioesterase domain containing protein, and the described NRPS gene in place, the composition of the *BIIRfg* gene cluster is consistent with the biosynthesis of the cyclic lipodepsipeptide BII-Rafflesfungin.

### BII-Rafflesfungin has anti-fungal activity

We tested the effect of BII-Rafflesfungin on the growth of *Candida albicans* strain (SC5314) using the Clinical and Laboratory Standards Institute (CLSI) guidelines [58]. The widely used antifungal amphotericin B was used as a positive control. Exponentially-growing *Candida albicans* cells were treated with either BII-Rafflesfungin or amphotericin B at different concentrations and the growth as measured by OD_600 nm_ was recorded after 48 h. Consistent with published data [58], amphotericin B completely inhibited the growth of *C. albicans* cells at 125 nM, BII-Rafflesfungin completely inhibited the growth of *C. albicans* cells at 32 μM (see Additional File 11: Figure S13).

Furthermore, we expanded the antifungal activity evaluation of BII-Rafflesfungin to other yeast strains and filamentous fungi; and the results are summarised in Table 4. The dose response curves are shown in supplementary (Additional file 12: Figure S14). BII-Rafflesfungin inhibited the growth of two additional strains of *Candida* (ATCC 10231 and ATCC 90028) and *Saccharomyces cerevisiae* (Additional file 12 Figure S14 (a) and Table 4). The IC_50_ for the *C. albicans* strains ATCC10231 and ATCC 90028 were 2.4 μM and 4.6 μM, respectively. For the *S. cerevisiae* strain (BY4741), the IC_50_ was 2.7 μM. BII-Rafflesfungin is a potent inhibitor of *Aspergillus fumigatus* growth, with an IC_50_ of 1.2 μM; however, it is 6-fold less active against *A. brasiliensis* (Additional file 12 Figure S14 (b).

**Table 4:** Biological activity of BII_Rafflesfungin

Fungal strain/Cell line	IC50, μM
*Candida albicans* ATCC 10231	2.4
*Candida albicans* ATCC 90028	4.6
*Saccharomyces cerevisiae* (BY4741)	2.7
*Aspergillus fumigatus* ATCC 46645	1.2
*Aspergillus brasiliensis* ATCC 16404	7.4
A549 lung carcinoma cells	16.5
HepG2 liver carcinoma cells	13.8

To determine whether BII-Rafflesfungin has cytocidal or cytostatic activity, we tested the ability of *Candida albicans* (SC5314) cells to recover after pre-treatment with either BII-Rafflesfungin (100 μM) or DMSO for 6 h. We used Amphotericin B (10 μM; a cytocidal inhibitor) and Itraconazole (100 μM; a cytostatic inhibitor) as controls. Treatment with all three compounds for 6 h completely inhibited the growth of yeast cells (Additional file 13: Figure S15 A). We then washed off the compound and transferred the cells onto drug-free YPD agar plates. As expected Itraconazole-treated cells were able to grow after their transfer to drug-free YPD agar plates (Additional file 13: Figure S15 B). Similar to cells treated with Amphotericin B, BII-Rafflesfungin-treated cells failed to grow on YPD agar plates indicating that BII-Rafflesfungin has cytocidal activity (Additional file 13: Figure S15 B).

We also evaluated the mammalian cytotoxicity of BII-Rafflesfungin against 2 established cell lines, A549 lung carcinoma cells and HepG2 liver carcinoma cells (Table 4; Fig. S14 (c)). BII-Rafflesfungin was cytotoxic against both cell lines with an IC_50_ of 16.5 μM and 13.8 μM, respectively.

To conclude, BII-Rafflesfungin has potent broad spectrum anti-fungal activity but moderate level of mammalian cytotoxicity.

## Discussion

With a continuous increase in the number of fungal genomes being sequenced, there is a substantial evidence about the abundance of various secondary metabolites which are synthesized by different NRPS/PKS gene clusters. Several of these fungal secondary metabolites are cyclic lipopeptides like destruxins [59], aureobasidine [60], beauvericine [61], apicidin [62] or echinocandins [35]. We have now identified another cyclic depsipeptide in a fungal genome, BII-Rafflesfungin, which is a novel analogue of Phomafungin [25]. We identified the NRPS-t1PKS gene cluster, which we name *BIIRfg*, responsible for the synthesis of BII-Rafflesfungin. The vicinity of *BIIRfg_NRPS* was explored to identify a polyketide synthase, *BIIRfg_PKS* gene, an AMP-dependent ligase, and a type II thioesterase (TEII) which completes the *BIIRfg* cluster for the biosynthesis of BII-Rafflesfungin. Of note, type II thioesterases are often found to be encoded within PKS and NRPS gene clusters; they fulfill diverse functions, including removal of aberrant residues blocking the megasynthase (repair function), and various roles in substrate selection or, release of intermediates and products [56, 57, 63].

Sequence analysis of *BIIRfg* identified ten modules consisting of 27 domains which were part of a single NRPS gene. The initial module M1 lacks the A domain as well as the C domain which is consistent with the other known NRPS clusters for lipopeptides like echinocandin (EcdA;AFT91378) [35, 37, 64], emericellamide (EasA; XP_660149) [36] and verlamelin (vlmS;AB862312) [38]. It has been reported that, in such cases, the initial module accepts the activated fatty acid intermediate at the PCP domain for further elongation of the actual peptide by downstream modules [37].

At the outset, we had no independent information about the structure of BII-Rafflesfungin. Since the specificities of many of the A domains have been biochemically characterized [11, 46, 65] and several prediction algorithms have been implemented to predict the specificity determining codes of A domains [34, 46, 66–72], it is possible to analyse the amino acid sequences of some A domains in order to predict which amino acid would be activated by the corresponding A domain. Therefore, we have investigated the sequences of all the A domains and tried to infer the specificity of binding amino acids from the signature sequence encoded in the A domains. The sequence segments representing A domains are actually Acyl-CoA synthetase NRPS A domains and belong to the Luciferase superfamily of enzymes [73]. Adenylation domains are organized two-partite: (i) a N-terminal core domain and (ii) a C-terminal sub-domain. Several characteristic conserved motifs of A domains (these are mA1:LTYxEL, mA2:LKAGxAYVPID, mA3:LAYxxYTSGTTGxPKG, mA4:FdxS, mA5:NxYGPTE, mA6:GELxIxGxGLARGYW, mA7:YKTGDQ, mA8:GrxDxQVKIRGxRVELEEVE, mA9:LpxYMIP and mA10:NGKIDR) have been well established in various studies [11, 74]. The positioning of the respective substrate α-amino acid is governed by the highly conserved aspartate residue in the A4 motif of the core domain and the lysine residue in the A10 motif of the sub-domain [75]. Originally, it was established that the approximately 100 amino acid residues in the region between core motifs A4 and A5 have ~ 10 critical residues (in the case of the gramicidin S synthetase (GrsA; P0C061), these 10 residues are: D235, A236, W239, T278, I299, A301, A322, I330, C331, also including K517), which represent the signature sequence of an A domain and determine its substrate specificity. But later, Challis et al. [46] explained that Cys331 and Lys 517 can be excluded from the signature sequence as Cys331 points away from the specificity pocket in GrsA [11] while Lys 517 is strictly conserved and does not participate in substrate discrimination. Hence, we took only eight residues in consideration as the signature sequence for the adenylation domains. This set of eight amino acids present at such specific locations is commonly referred to as Stachelhaus code [11]. The sequence positions of these codes are determined based on their proximity to the conserved sequence motifs mA4 and mA5.

The collinearity rule for NRPS genes implies that the order of different modules of an NRPS is coherent with the amino acid sequence of the corresponding peptide product [76]. However certain exceptions/variations to this rule have already been reported [77, 78]. The Stachelhaus codes from the A domains’ sequence for *BIIRfg_NRPS* combined with the collinearity rule helped to predict some A domain – amino acid assignments. The presence of identical signature codes in A4, A5, and A6 coincides with the observation of three serine residues in BII-Rafflesfungin. Two other codes (for A2 and A7) were predicted based on a sequence similarity approach and these predictions were later confirmed by HRESIMS data.

The original antiSMASH analysis left a C domain in the second last module missing. This domain was later identified using a HMMER search (see details in the Results section and in Additional file 3: Figure S2) which completed the *BIIRfg* cluster. The list of C domains belonging to the NRPS gene therefore appears to be complete. Various studies on the C domain phylogeny [49, 79, 80] illustrated that these condensation domains have different functional subtypes like:

(i) the ^L^C_L_ (donor C acceptor) domain (it catalyses a peptide bond between two L-amino acids),
(ii) the ^D^C_L_ domain (it catalyses a peptide bond to link a L-amino acid to the elongation peptide ending with a D-amino acids),
(iii) the starter C Domain (it catalyses the acylation of the first amino acid with a β-hydroxyl-carboxylic acid) and,
(iv) the dual E/C domain (it catalyses the peptide bond formation and cyclization of cysteine (Cys), serine (Ser) and threonine (Thr) residues.

There are no clear sequence distinctions between ^L^C_D_ or ^D^C_D_ type of condensation domains [79]. Despite being best scored by the ^D^C_L_ HMMs (Additional file 5: Table S2), the ML tree clusters the C domains from *BIIRfg_NRPS* with the dual type which however is very close to the ^D^C_L_ type. Similar pattern in clustering of ^D^C_L_ and dual type domains along with other types of C domain have been reported earlier [80] suggesting that these two types of domains are closely related. In fact, biochemical experiments had provided insights that dual C domains have catalytic roles for condensation and epimerization and act as ^D^C_L_ catalysts [81].

Although all the C domains in *BIIRfg_NRPS* cluster with the dual type, their amino acid sequences do not show the presence of the ‘HHI/LxxxxGD’ motif which is known to be present in the N terminal region of dual C domains along with the ‘HHxxxDG’ motif [49, 81]. The presence of these different core motifs in the C domains was first described by Crecy-Lagard et al. [82] and Marahiel et al. [51] and was subsequently updated by Rausch et al. [49]. As can be seen from the alignment (Fig. 3), all the C domains (with the exception of C1, C7, C8 and C9 (C_T_)) harbor the conserved ‘HHxxxDG’ motif; C1 and C9 have a ‘SHxxxDG’ motif while C7 and C8 show the presence of an alanine residue in place of the usual glycine residue in a ‘SHxxxDA’ motif. The second His-residue was found conserved in all these condensation domains which is known to be essential for the catalytic function of these domains [83, 84].

Based on all the genomic and experimental information, it is still not trivial to predict the stereochemistry of the amino acids in the final peptide. Nevertheless, it has been reported that traditionally E domains are associated with the modules which add D-amino acids in final peptides like surfactin [85], verlamelin [38] and other cyclic lipopeptides like bacillomycin, locillomycin and fengycin [86]. The epimerization domains in the *BIIRfg_NRPS* gene observed here also harbor the same ‘HHxxxDxVSW’ motif (where the second histidine is functionally important) [49, 79, 87, 88] (see alignment in Additional File 7: Figure S9). In the case of BII-Rafflesfungin, we were able to show that Ser and allo-Thr that are recognized by A6 (M7) and A7 (M8) are in D-configuration; i.e., there are D-Ser6 and D-allo-Thr7 in the peptide product.

### Biosynthesis of BII-Rafflesfungin

**Fig. 6:**
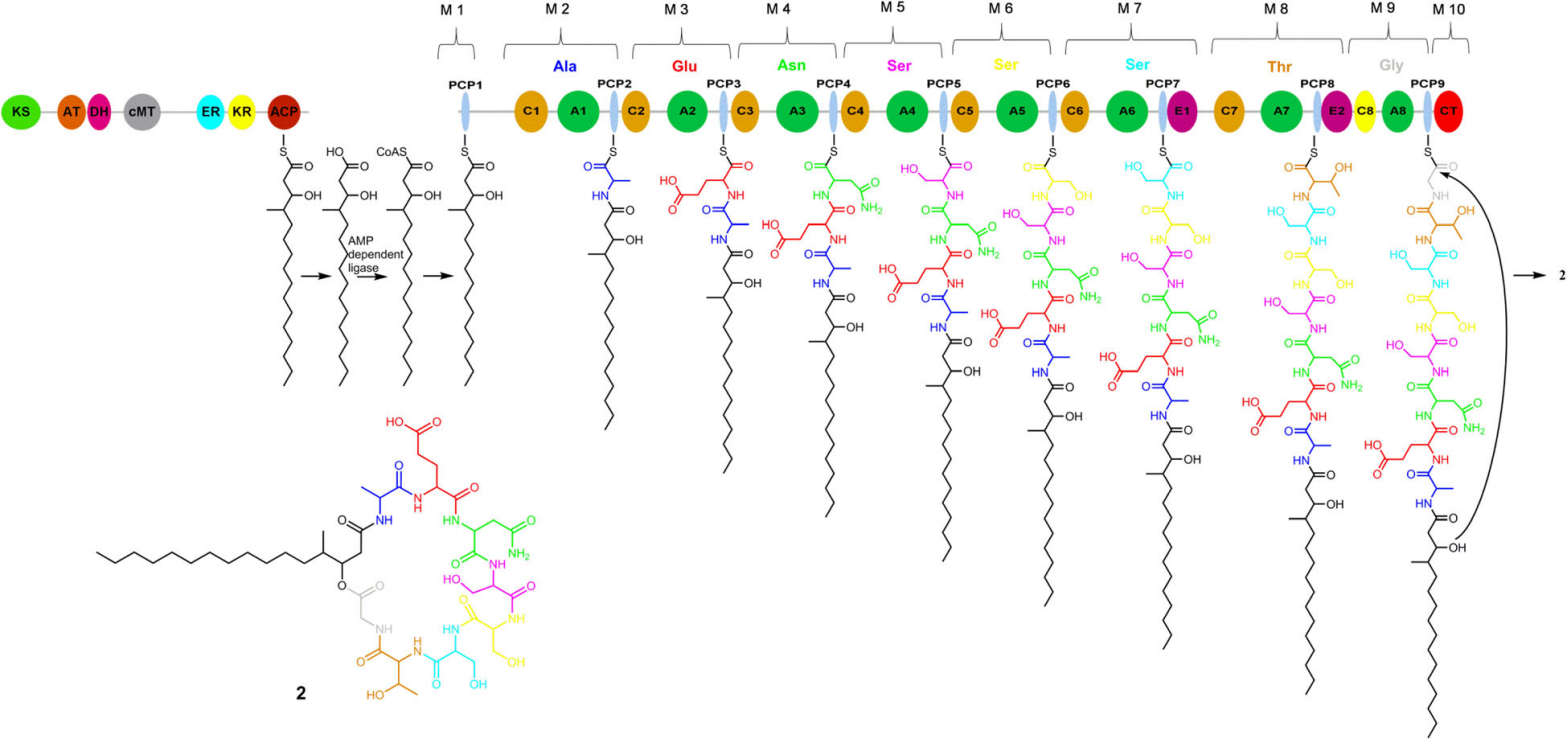
Proposed biosynthetic pathway for the synthesis of BII-Rafflesfungin

Based on our combined computational and experimental results, we propose the following mechanism for the biosynthesis of BII-Rafflesfungin: The lipid part of the compound, β-hydroxy-γ-methyl hexadecanoic acid (HMHDA), is assembled by the *BIIRfg_PKS* cluster. The cluster itself contains all the necessary domains including a methyltransferase. A similar scenario was described for the biosynthesis of the fungal compound pneumocandin where the present PKS is thought to be responsible for the synthesis of the dimethylmyristate side chain [37, 89]. Based on the current knowledge from different pathways such as echinocandin B, emericellamide, pneumocandin and verlamelin biosynthesis [37, 38, 64, 89], we propose that the lipid moiety is released from the PKS module and activated to form an acyladenylate by orf-i, the predicted AMP-dependent ligase, and, subsequently, loaded on to the first PCP domain of the *BIIRfg_NRPS* gene to initiate the peptide synthesis as shown in Fig. 6. The C_T_ domain, the last condensation domain of *BIIRfg_NRPS,* acts like a type I thioesterase. It terminates the peptide synthesis and releases the cyclic lipodepsipeptide BII-Rafflesfungin. We can only speculate on the function of the predicted thioesterase II, in many cases, it has a repair function by removing aberrant residues blocking the megasynthase [63].

### Mechanism of action of BII-Rafflesfungin

BII-Rafflesfungin displayed antifungal activity against *Candida albicans*, *Saccharomyces cerevisiae* and *Aspergillus fumigatus*. BII-Rafflesfungin’s cytotoxic mode of inhibition suggests that it perturbs the membrane integrity of yeast cells. Similar mode of action was also proposed for Phomafungin, based on its *C. albicans* fitness test profile [25]. The long hydrophobic chain may facilitate its insertion into the plasma membrane and the polar cyclic peptide unit might affect the membrane structure. For Phomafungin, it was speculated that the primary effect on the plasma membrane is affected by changes in sphingolipid content and Ca^2+^ concentration [25]. Interestingly, cyclic depsipeptides Syringomycin and Syringopeptins from *Pseudomonas* inhibit the growth of yeast cells by binding to the plasma membrane [90–92]. Another cyclic depsipeptide, Aureobasidin, produced by the fungus *Aureobasidium pullulan* affects membrane structure by inhibiting inositol phosphorylceramide (IPC, sphingolipid) synthase, a key enzyme involved in sphingolipid biosynthesis [93]. Chemogenomic profiling, a fitness-based assay involving barcoded deletion strains in yeast, could give insights into BII-Rafflesfungin’s mode-of -action [94].

## Conclusions

We have discovered a biosynthetic gene cluster predicted to synthesize BII-Rafflesfungin, which is a novel analogue of the known compound Phomafungin. The two compounds have the same amino acid composition with the exception of the presence of two homo-serine residues in Phomafungin instead of two common serines in BII-Rafflesfungin. This is the first study stating the presence of a NRPS-t1PKS gene cluster in *Phoma sp* for the synthesis of a novel analogue of Phomafungin. The compound BII-Rafflesfungin is biologically important as it shows antifungal activity.

The NRPS-t1PKS cluster ‘*BIIRfg’* was found to be complete by the software tool antiSMASH except one C domain in the second last module of *BIIRfg_NRPS* gene and one ACP domain in the *BIIRfg_PKS* gene which were later identified by in-depth sequence analysis. The order of the amino acids in the final peptide product was experimentally verified using NMR studies and novel specificity conferring codes were predicted. Further studies using the available biochemical assays [95–99] to confirm the amino acid specificity for the A domains would be insightful. The mechanism of synthesis of BII-Rafflesfungin is proposed which involves the formation of the lipid part by *BIIRfg_PKS* followed by activation and transfer of the lipid chain by the predicted AMP-ligase on to the first PCP domain of *BIIRfg_NRPS* gene. This initiates the peptide synthesis by *BIIRfg_NRPS* gene which successively add different amino acids to the growing peptide and in the end releases the final cyclic lipodepsipeptide, BII-Rafflesfungin.

## Methods

### Fungal strain

The fungal strain was isolated from a black sea cucumber near the coastal area of Raffles Marina Clubhouse, Singapore. The strain was initially numbered as F3723. The collected black sea cucumber was blended and particles of size 105–210 μm were resuspended in sterile water. Serial dilutions of this suspension were then plated on marine agar supplemented with 50 mg/L tetracycline and allowed to grow at 24 °C for up to 30 days. Fungal colonies growing on the isolation plates were sub-cultured onto marine agar to obtain pure cultures. F3723 was maintained as frozen mycelia in 10% glycerol at -80 °C.

### DNA isolation and sequencing

F3723 was grown for 5 days at 24 °C in YMG media (10 g/L malt extract, 4 g/L yeast extract and 4 g/L glucose), and the mycelia were harvested by centrifugation at 4000 g for 10 min. High-quality and molecular weight genomic DNA (> 20 kb) was obtained using the modified CTAB method [100]. Cells were re-suspended in 2X CTAB buffer containing 2% hexadecyltrimethylammonium bromide (CTAB; Sigma), 1.5 M NaCl, 25 mM EDTA, 100 mM Tris-HCl pH 8.8 and 0.1% polyvinylpyrrolidone (Sigma) and homogenised. Proteins were then removed using 1% SDS and proteinase K (25 mg/mL) before treated with RNaseA (10 mg/mL) for the removal of RNA from the total nucleic acids. The DNA was further purified with 25:24:1 phenol chloroform isoamyl alcohol (Sigma), followed by 24:1 chloroform isoamyl alcohol (Sigma). Extracted DNA was re-suspended in 50 μL 10 mM TE buffer pH 8.0 and additional purification step was carried out using Mag-Bind® RxnPure Plus (OMEGA bio-tek) as per manufacturer’s protocol. The quality of DNA was verified using a NanoDrop^Tm^ 2000 spectrophotometer (Thermo Scientific), Qubit™fluorometric quantitation with Qubit™ 3.0 Fluorometer (Thermo Scientific) and agarose gel electrophoresis. Purified genomic DNA was sheared to approximately 20 kb using a g-Tube (Covaris). A SMRTbell library was prepared according to manufacturer’s instructions, loaded with a MagBead bound library protocol onto two SMRTCells at 0.125 and 0.3 nM, and sequenced using the P5-C3 chemistry on the PacBio RSII instrument (Pacific Biosciences) with a 180 min movie time. Two additional SMRTCells were run at 0.2 and 0.4 nM and sequenced using the P6-C4 chemistry on the PacBio RSII instrument with a 240 min movie time. De novo assembly was performed with the Hierarchical Genome Assembly Process 3 (HGAP3) [101] in the SMRT Analysis suite (version 2.3) using all default parameters. In total, 288,789 reads were collected, with a mean read length of 13,441 bp, giving a total of 3,881,628,628 bp of raw sequence. De novo assembly of the raw sequence resulted in 122 polished contigs. The total length of these 122 assembled contigs was 35,411,911 bp; the largest contig size was 2,158,956 bp, and the N50 contig length was 978,069 bp. Given the final genome assembly size, therefore, the overall sequencing coverage was ~ 110 × .

### BII-Rafflesfungin: extraction and isolation

The culture broths (160 × 50 mL, total 8 L) of *Phoma* (F3723) were combined and centrifuged to separate the supernatant and the mycelia. The combined mycelia were freeze-dried, extracted two times with MeOH (4 L), filtered and, then, were evaporated to dryness using rotary evaporation. One litre of water was added to the dried methanolic extract (94 g) and charged to a 10 cm × 8 cm Sepra C18-E (50 μm, 65A, phenomenex) column. The column was eluted by isocratic gradient of 20, 50, 80, and 100% aqueous methanol. Each fraction was submitted to antifungal activity testing. The antifungal activity was found to be concentrated in the 80% aqueous methanol fraction, which was then concentrated under reduced pressure and yielded 0.25 g of a partially enriched fraction. The 80% aqueous methanol dried extract was dissolved in 2.5 mL of DMSO and separated by C18 reversed-phase preparative HPLC (solvent A: H_2_O + 0.1% HCOOH, solvent B: ACN + 0.1% HCOOH; flow rate: 30 mL/min, gradient conditions: 65:35 isocratic for 5 min; 35 to 70% of solvent B over 85 min, followed by 70 to 100% of solvent B over 10 min, and finally isocratic at 100% solvent B for 10 min to give BII-Rafflesfungin (**2**, 10 mg).

### Chemical structural data

The ^1^H, ^13^C, HSQC, COSY, and HMBC spectra of the compound are provided in Additional file 4: Figures. S3 to S7.

BII-Rafflesfungin (2) yellowish oil; [α]_D_ + 65.3 (c 0.2, MeOH); UV (MeOH) λ_max_ (log ε) end absorption nm; HRESIMS m/z 1002.5371 (calcd for C_44_H_75_N_9_O_17_ + H, 1002.5354); ^1^H and ^13^C NMR data, see Table 2.

### General experimental procedures

JASCO P-2000 digital polarimeter was used to record the optical rotations while GE Healthcare Ultrospec 9000 spectrophotometer was used to obtain the UV spectra.

Bruker DRX-400 NMR spectrometer with Cryoprobe was used to collect the NMR spectra. 5-mm BBI (^1^H, G-COSY, multiplicity-edited G-HSQC, and G-HMBC spectra) or BBO (^13^C spectra) probe heads equipped with z-gradients were used.

Agilent 1260 Infinity Preparative-Scale LC/MS Purification System and Agilent 6130B single quadrupole mass spectrometer for LC and LC/MS Systems were used to perform the preparative HPLC analysis.

Agilent UHPLC 1290 Infinity coupled to Agilent 6540 accurate-mass quadrupole time-of-flight (QTOF) mass spectrometer which was equipped with a splitter and an ESI source were used to acquire the HRESIMS and MS/MS spectra. For over 15 min, under standard gradient condition of 100% water with 0.1% formic acid to 100% acetonitrile with 0.1% formic acid, the analysis was performed with a C18 4.6 × 75 mm, 2.7 μm column at flowrate of 2 mL/min. The operating parameters for QTOF were the same as in [6].

Nα-(2,4-Dinitro-5-fluorophenyl)-L-alaninamide (L-FDAA) and amino acid standards were purchased from Sigma Aldrich except D-allo-threonine which was purchased from Chem Cruz. Aspartic acid and glutamic acid were converted from asparagine and glutamine (Sigma Aldrich), respectively.

### Hydrolysis of BII-Rafflesfungin

To a stirred solution of BII-Rafflesfungin (**2**, 1 mg) and 1 mL of distilled water, 1 mL of 0.01 M NaOH was added. The mixture was stirred for 19 h at room temperature. The reaction mixture was charged on a small column of C-18 silica gel and washed with water, and the linear peptide was then eluted with MeOH. The MeOH fraction was concentrated and dried using rotary evaporator to yield 0.8 mg of the opened form of the linear peptide, as light yellowish oil. HRESIMS m/z 1020.5470 (calcd for C_44_H_77_N_9_O_18_ + H, 1020.5459). HRESIMS/MS: m/z 945.5151 (calcd for C_42_H_73_N_8_O_16_, 945.5139), 844.4669 (calcd for C_38_H_66_N_7_O_14_, 844.4662), 757.4343 (calcd for C_35_H_61_N_6_O_12_, 757.4342), 681.2686 (calcd for C_24_H_41_N_8_O_15_, 681.2686), 670.4028 (calcd for C_3_2H_56_N_5_O_10_, 670.4022), 583.3701 (calcd for C_29_H_51_N_4_O_8_, 583.3701), 552.2255 (calcd for C_19_H_34_N_7_O_12_, 552.2260), 469.3270 (calcd for C_25_H_45_N_2_O_6_, 469.3272), 438.1828 (calcd for C_15_H_28_N_5_O_10_, 438.1831), 340.2844 (calcd for C_20_H_38_NO_3_, 340.2846).

### Acid hydrolysis and derivatization of BII-Rafflesfungin

BII-Rafflesfungin (0.4 mg) was dissolved in 0.5 mL of 6 N HCl and heated at 100 °C for 8 h. The reaction mixture was cooled to room temperature and evaporated to dryness. The dried acid hydrolysate was added in 100 μL of H_2_O, 100 μL of 1% Nα-(2,4-Dinitro-5-fluorophenyl)-L-alaninamide (FDAA) in acetone, and 40 μL of 1 M sodium bicarbonate. The mixture was heated at 40 °C, with stirring for 1 h. The reaction mixture was then cooled to room temperature, neutralized with 20 μL of 2 M HCl, and dried in vacuo. The residue was dissolved in H_2_O (200 μL) and centrifuged to remove insoluble material before LCMS analysis [Zorbax Eclipse Plus 2.1 × 50 mm 1.8 μm, elution with 20 min linear gradient of 10–45% of solvent B (ACN 0.1% FA)]. The retention time (t_R_, min) for the standard amino acids DAA-amino acid derivatives were as follows: L-alanine (8.661), D-alanine (10.377), L-serine (6.379), D-serine (6.660), glycine (7.922), L-glutamic acid (7.831), D-glutamic acid (8.807), L-aspartic acid (7.100), D-aspartic acid (7.900), L-threonine (6.775), D-threonine (8.816), L-allo-threonine (6.941), D-allo-threonine (7.845) (Additional file 8: Figure S10).

### Species annotation and biosynthetic cluster detection

Blast [102] based taxonomic analysis of the 18S rRNA was done which supports the species annotation of F3723. In addition, the beta-tubulin gene from F3723 was scanned against NT database using a blastn search. The most similar sequences were selected and incorporated into a multiple sequence alignment using ClustalO [103]. A maximum likelihood tree was generated from the alignment using Mega7 with JTT matrix under discrete gamma distribution with four categories [50].

The assembled *Phoma* genome was analysed by a locally installed antiSMASH (version 3.0.3) [34, 104]. Protein coding genes were predicted by using AUGUSTUS (version 3.2.2, default parameters with *Fusarium graminearum* as training set) [30] and GeneMark-ES (version4.10, default parameters) [31, 32]. The predicted protein sets were further analysed by ANNOTATOR tools [33]. Manual annotation efforts were helpful to detect missing function predictions.

### Phylogenetic analysis of the C domains

All the sequences of the different subtypes of C domains were obtained from the NaPDoS server [80]. The amino acid sequences of all C domains from *BIIRfg* cluster were aligned with the NaPDos dataset using MUSCLE [105]. The ML tree was constructed using Mega7 with JTT matrix under discrete gamma distribution with four categories [50].

### Antifungal assays

The Minimal Inhibitory Concentration (MIC) of *C. albicans* (SC5314) cells was determined using the CLSI guidelines [58]. Cells were treated with various concentrations (0 μM – 64 μM, see Additional file 11: Figure S13) of BII-Rafflesfungin and Amphotericin B (0 nM – 1000 nM, Fig. S13) in RPMI 1640 medium in duplicates in a 96-well microplate. The microplate was photographed after 48 h of incubation at 35 °C. The least concentration of the compound that caused an optically clear well was computed as its MIC.

Antifungal activity was also determined against five strains: three yeasts, *Candida albicans* ATCC 10231, *Candida albicans* ATCC 90028 and *Saccharomyces cerevisiae* BY4741; and two moulds, *Aspergillus brasiliensis* ATCC 16404 and *Aspergillus fumigatus* ATCC 46645. The activity was evaluated by determining the IC50 value using a rapid microbroth dilution method in 96- or 384-well microplate format [106]. Logarithmically growing yeast (*Candida albicans*/ *Saccharomyces cerevisiae)* cells in Sabouraud Dextrose Broth (SDB) medium were treated with various concentrations (0, 0.47, 0.94, 1.88, 3.75, 7.5, 15 and 30 μM) of BII-Rafflesfungin in triplicates. Cultures of *Candida albicans* strains ATCC 10231 and ATCC 90028 were incubated at 25 °C and 35 °C, respectively. *Saccharomyces cerevisiae* cultures were incubated at 30 °C. The growth (OD_600nm_) was recorded after 24 h incubation.

Frozen stocks of *Aspergillus* conidial suspensions were diluted in SDB medium to final concentrations of approximately 2.5 × 103 spores/mL (ATCC 16404) or 2.5 × 104 spores/mL (ATCC 46645). These conidial suspensions were then added to various concentrations (3 nM to 100 μM) of BII-Rafflesfungin in 384-well microplates in triplicates. The microplates were subsequently incubated for 72 h at 24 °C prior to OD_600nm_ measurement.

For the cytocidal/cytostatic activity test, *C. albicans* (SC5314) cells at a starting OD_600 nm_ of 0.6 were incubated with either DMSO or BII-Rafflesfungin (100 μM) or Amphotericin B (10 μM) or Itraconazole (100 μM) in duplicate in YPD medium at 30 °C with shaking (220 rpm). OD_600nm_ values of the cultures after 6 h were obtained. Subsequently, the cells were washed twice, normalized and diluted in YPD medium. Equal volumes (3 μl) were spotted on YPD agar plates. Growth was recorded after incubation of the plates at 30 °C for 2 days.

Cytotoxicity of BII-Rafflesfungin on the A549 human lung carcinoma cell line and the liver carcinoma cell line HepG2 were determined as described before [6]. A549 cells were seeded at 1500 cells per well, and HepG2 cells were seeded at 2500 cells per well in a 384-well microplate. Cells were treated with a series of BII-Rafflesfungin concentrations ranging from 0.8 μM to 100 μM and incubated for 72 h at 37 °C in the presence of 5% CO2. Cytotoxic effect of the compound was measured using the PrestoBlue™ cell viability reagent (Life Technologies). Following incubation of the microplates with the dye for 2 h, the fluorescence reading (Excitation / Emission: 560 nm /590 nm) was recorded using the Tecan Infinite M1000 Pro reader.

## Additional files

Additional file 1: **Table S1.** antiSMASH results (links) for publicly available full genome assemblies of *Phoma* sp. in NCBI (Feb 2019). (PDF 22 kb)

Additional file 2: **Figure S1.** Circular representation of the phylogenetic tree of protein coding marker beta-tubulin for inferring the taxonomic classification of strain F3723. (PDF 147 kb)

Additional file 3: **Figure S2.** Prediction of the missing C domain in Module 9. (PDF 551 kb)

Additional file 4: **Figure S3.** ^1^H NMR spectrum (DMSO-d6, 400 MHz) of BII-Rafflesfungin. **Figure S4.** ^13^C NMR spectrum (DMSO-*d*_6_, 100 MHz) of BII-Rafflesfungin. **Figure S5.** HSQC spectrum (DMSO-*d*_6_, 400 MHz) of BII-Rafflesfungin. **Figure S6.** COSY spectrum (DMSO-*d*_6_, 400 MHz) of BII-Rafflesfungin. **Figure S7.** HMBC spectrum (DMSO-*d*_6_, 400 MHz) of BII-Rafflesfungin. (PDF 196 kb)

Additional file 5: **Table S2.** HMMER search results against the HMM models of ^D^C_L_, ^L^C_L_, starter and dual type of C domains with all 9 C domains as query sequences. (PDF 78 kb)

Additional file 6: **Figure S8.** Phylogenetic tree analysis of the functional distribution of C domains using MEGA7 (Maximum Likelihood (ML) tree). (PDF 23 kb)

Additional file 7: **Figure S9.** Multiple sequence alignment of the two epimerization domains E1 and E2 from the *BIIRfg_NRPS* gene. (PDF 124 kb)

Additional file 8: **Figure S10.** Results of the LCMS analysis of the absolute configuration of BII-Rafflesfungin by Marfey’s method. (PDF 111 kb)

Additional file 9: **Figure S11.** Multiple sequence alignment of the predicted AMP-dependent ligase for BII-Rafflesfungin biosynthesis along with VlmC (AMP-dependent ligase from Verlamelin biosynthesis) and EcdI (AMP-dependent ligase from Echinocandin B biosynthesis). (PDF 353 kb)

Additional file 10: **Figure S12.** Multiple sequence alignment of the predicted Type II thioesterase (orf-a) along with the known TEII of Surfactin-Synthetase (SrfAD) and Rifamycin (RifR). (PDF 215 kb)

Additional file 11: **Figure S13.** BII-Rafflesfungin inhibits the growth of yeast cells/standard CLSI test with Amphotericin B as positive control. (PDF 224 kb)

Additional file 12: **Figure S14.** Growth inhibitory effects of BII Rafflesfungin against yeasts (a), Aspergillus species (b) and mammalian cell lines (c). (PDF 100 kb)

Additional file 13: **Figure S15.** BII-Rafflesfungin has cytocidal activity. (PDF 40 kb)
