# Supplementary material for: Genomics-driven discovery of a biosynthetic gene cluster required for the synthesis of BII-Rafflesfungin from the fungus Phoma sp. F3723
Source: BMC Genomics. 2019 May 14;20:374. doi: 10.1186/s12864-019-5762-6 (PMC6518819; doi:10.1186/s12864-019-5762-6)
Supplement: Supplementary file 1 — Table S1. antiSMASH results (links) for publicly available full genome assemblies of Phoma sp. in NCBI (Feb 2019). (PDF 22 kb) [file 12864_2019_5762_MOESM1_ESM.pdf]

Table S1: antiSMASH results for publicly available full genome assemblies of *Phoma* sp. in NCBI (Feb 2019).

Species	Accession number	
Phoma herbarum	GCA_001599375.1	
https://fungismash.secondarymetabolites.org/upload/fungi-bfd8ee4b-2d68-4d85-ae11-d4fcca2bf1c1/index.html		
Phoma koolunga	GCA_004151575.1	
https://fungismash.secondarymetabolites.org/upload/fungi-fb1774d6-1352-4ae6-b2c1-69e101f6c2a5/index.html		
Phoma koolunga	GCA_004151145.1	
https://fungismash.secondarymetabolites.org/upload/fungi-fb080fce-3824-4c69-82e1-40b66a3a89d1/index.html		
Phoma koolunga	GCA_004151165.1	
https://fungismash.secondarymetabolites.org/upload/fungi-ec1b5e37-13ca-42d5-8b4b-b195dedc1485/index.html		

Initially, there was only one publicly available full genome assembly of *Phoma* in NCBI. The assembly belongs to *Phoma herbarum (*GenBank assembly accession: GCA_001599375.1, Genome coverage:462x; Genome size: 39.26 MB; Scaffolds: 218 and Contigs:498). We have run antiSMASH webserver for fungal sequence to detect any potential biosynthetic cluster but the results do not show the presence of any complete cluster which could be predicted to synthesize Phomafungin.
The antiSMASH run results could be found at the given link:

https://fungismash.secondarymetabolites.org/upload/fungi-bfd8ee4b-2d68-4d85-ae11-d4fcca2bf1c1/index.html

Recently in 2019, three more assemblies of *Phoma koolunga were submitted to NCBI.* We have performed the antiSMASH analysis of all these assemblies. The links to the antiSMASH run aregiven below. Similar to *Phoma herbarum* genome, we do not see the presence of any complete cluster in these genomes as well.

1. *Phoma koolunga* (Genbak accession number: GCA_004151575.1, Genome coverage: 70.0x; Genome size: 50.29 MB, contigs:27,692) https://fungismash.secondarymetabolites.org/upload/fungi-fb1774d6-1352-4ae6-b2c1-69e101f6c2a5/index.html

2. *Phoma koolunga* (Genbak accession number: GCA_004151145.1, Genome coverage: 40.0x; Genome size: 43.76 MB, contigs:23,210) https://fungismash.secondarymetabolites.org/upload/fungi-fb080fce-3824-4c69-82e1-40b66a3a89d1/index.html

3. *Phoma koolunga* (Genbak accession number: GCA_004151165.1, Genome coverage: 140.0x; Genome size: 50.46 MB, contigs:21,3995) https://fungismash.secondarymetabolites.org/upload/fungi-ec1b5e37-13ca-42d5-8b4b-b195dedc1485/index.html
