# Supplementary material for: Genomics-driven discovery of a biosynthetic gene cluster required for the synthesis of BII-Rafflesfungin from the fungus Phoma sp. F3723
Source: BMC Genomics. 2019 May 14;20:374. doi: 10.1186/s12864-019-5762-6 (PMC6518819; doi:10.1186/s12864-019-5762-6)
Supplement: Supplementary file 2 — Figure S1. Circular representation of the phylogenetic tree of protein coding marker beta-tubulin for inferring the taxonomic classification of strain F3723. (PDF 147 kb) [file 12864_2019_5762_MOESM2_ESM.pdf]

Figure: S1: Circular representation of the phylogenetic tree of protein coding marker beta-tubulin for inferring the taxonomic classification of strain F3723. F3723 clusters together with all other *Phoma* species which are highlighted in red circle markers.

The evolutionary history was inferred by using the Maximum Likelihood method based on the Jukes-Cantor model [1]. The tree with the highest log likelihood (-7643.5657) is shown. Initial tree(s) for the heuristic search were obtained automatically by applying Neighbor-Join and BioNJ algorithms to a matrix of pairwise distances estimated using the Maximum Composite Likelihood (MCL) approach, and then selecting the topology with superior log likelihood value. A discrete Gamma distribution was used to model evolutionary rate differences among sites (4 categories (+*G*, parameter = 0.2124)). The tree is drawn to scale, with branch lengths measured in the number of substitutions per site. The analysis involved 51 nucleotide sequences. All positions with less than 95% site coverage were eliminated. That is, fewer than 5% alignment gaps, missing data, and ambiguous bases were allowed at any position. There were a total of 1146 positions in the final dataset. Evolutionary analyses were conducted in MEGA7 [2].

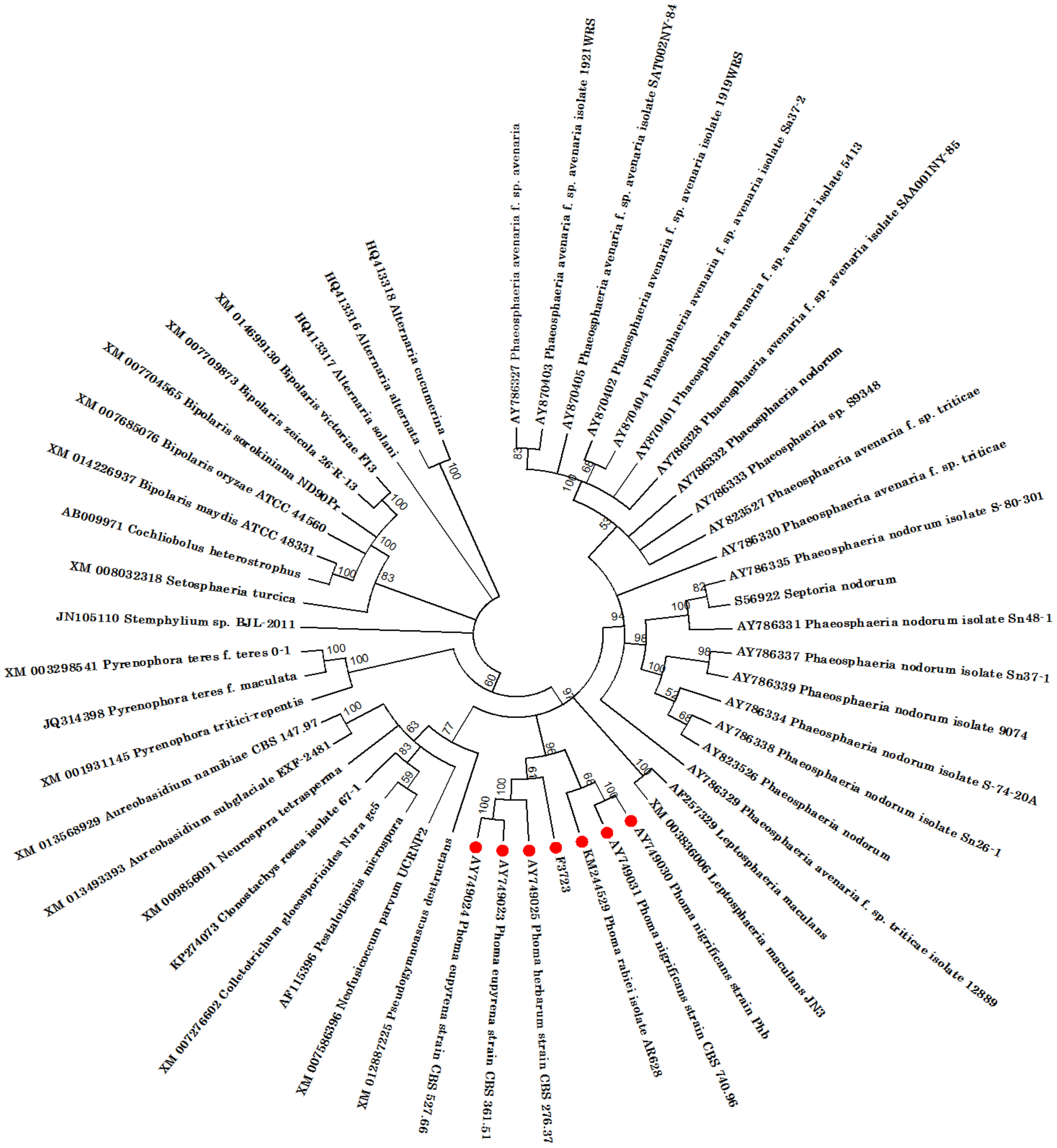

References

1. Jones D.T., Taylor W.R., and Thornton J.M. (1992**). The rapid generation of mutation data matrices from protein sequences**. Computer Applications in the Biosciences 8: 275-282.

2. Kumar S., Stecher G., and Tamura K. (2015**). MEGA7: Molecular Evolutionary Genetics Analysis version 7.0 for bigger datasets**. Molecular Biology and Evolution. Mol Biol Evol. 2016 Jul;33(7):1870-4.
