# Supplementary material for: Genomics-driven discovery of a biosynthetic gene cluster required for the synthesis of BII-Rafflesfungin from the fungus Phoma sp. F3723
Source: BMC Genomics. 2019 May 14;20:374. doi: 10.1186/s12864-019-5762-6 (PMC6518819; doi:10.1186/s12864-019-5762-6)
Supplement: Supplementary file 3 — Figure S2. Prediction of the missing C domain in Module 9. (PDF 551 kb) [file 12864_2019_5762_MOESM3_ESM.pdf]

Figure S2: Prediction of the missing C domain in Module 9

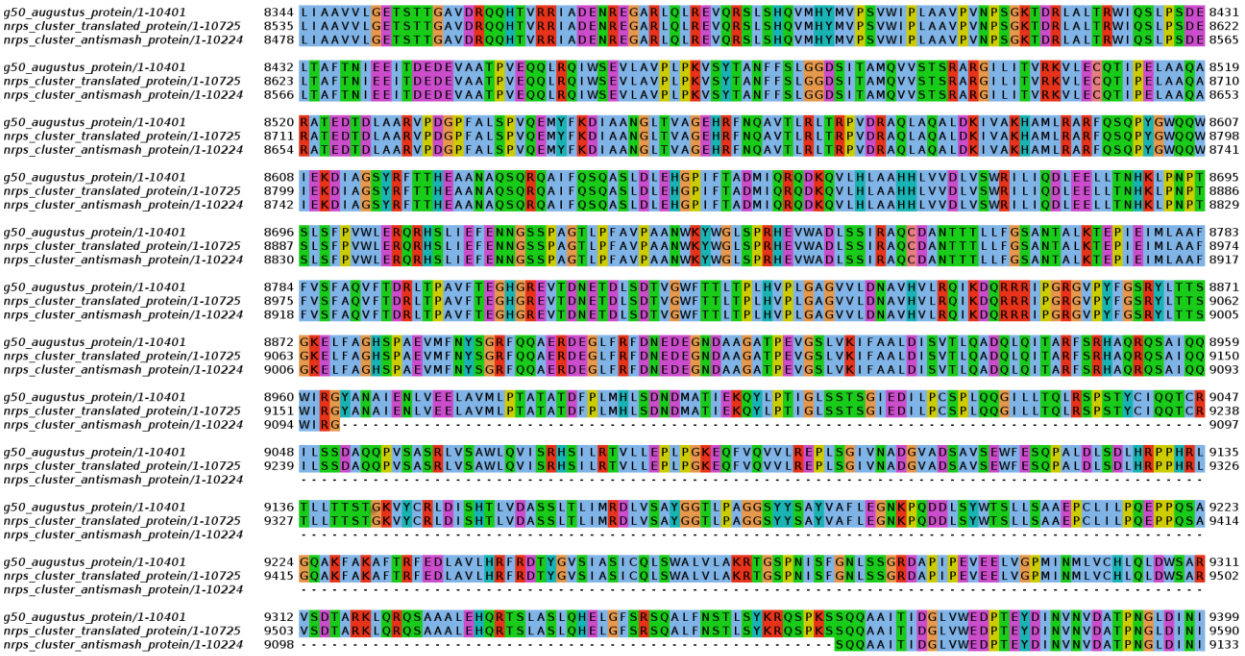

Direct analysis of the contig sequences with antiSMASH detected all the expected domains for the predicted gene cluster but, suspiciously, the C domain for the second last module (M9) was absent despite efforts to loosen parameters. We were, however, able to identify this missing C domain using profile-based search on the actual (translated) nucleic acid sequence. It appears that the antiSMASH algorithm identified a certain contig region potentially wrongly as intron. So, there were no domain predictions on the stretch of the sequence between E2 and A8 domains. The protein sequence of the NRPS gene region derived from the cluster contigs by antiSMASH have a length of 10224 amino acid residues, the one directly translated from the original contig was 10725 residues long. There was a difference of 501 residues (amino acid positions 9155 to 9554) shown in the alignment below. The next obvious step was to see whether this stretch codes for the missing condensation domain. We created the HMM profiles of all the detected C domains and scanned these profiles across the whole protein sequence of the NRPS cluster of 10725 amino acid residues. As anticipated, the same stretch 9155-9554 was a hit (alignment length 290 residue positions) with best C domain model scoring 263.5 (E-value=8.5e-82) using hmmsearch from HMMER3. We propose that these 290 aligned amino acids represent the C domain C8 for the second last module (M9).

The figure shows a part of the complete alignment from the protein sequences of the predicted NRPS cluster from antiSMASH, translated sequence of the same cluster using standard genetic code and predicted protein sequence of the same region from AUGUSTUS. The missing C domain sequence region predicted as intron by antiSMASH is shown as gaps in the alignment.
