# Supplementary material for: Genomics-driven discovery of a biosynthetic gene cluster required for the synthesis of BII-Rafflesfungin from the fungus Phoma sp. F3723
Source: BMC Genomics. 2019 May 14;20:374. doi: 10.1186/s12864-019-5762-6 (PMC6518819; doi:10.1186/s12864-019-5762-6)

Figure S3: ^{1}H NMR spectrum (DMSO-d_6, 400 MHz) of BII-Rafflesfungin

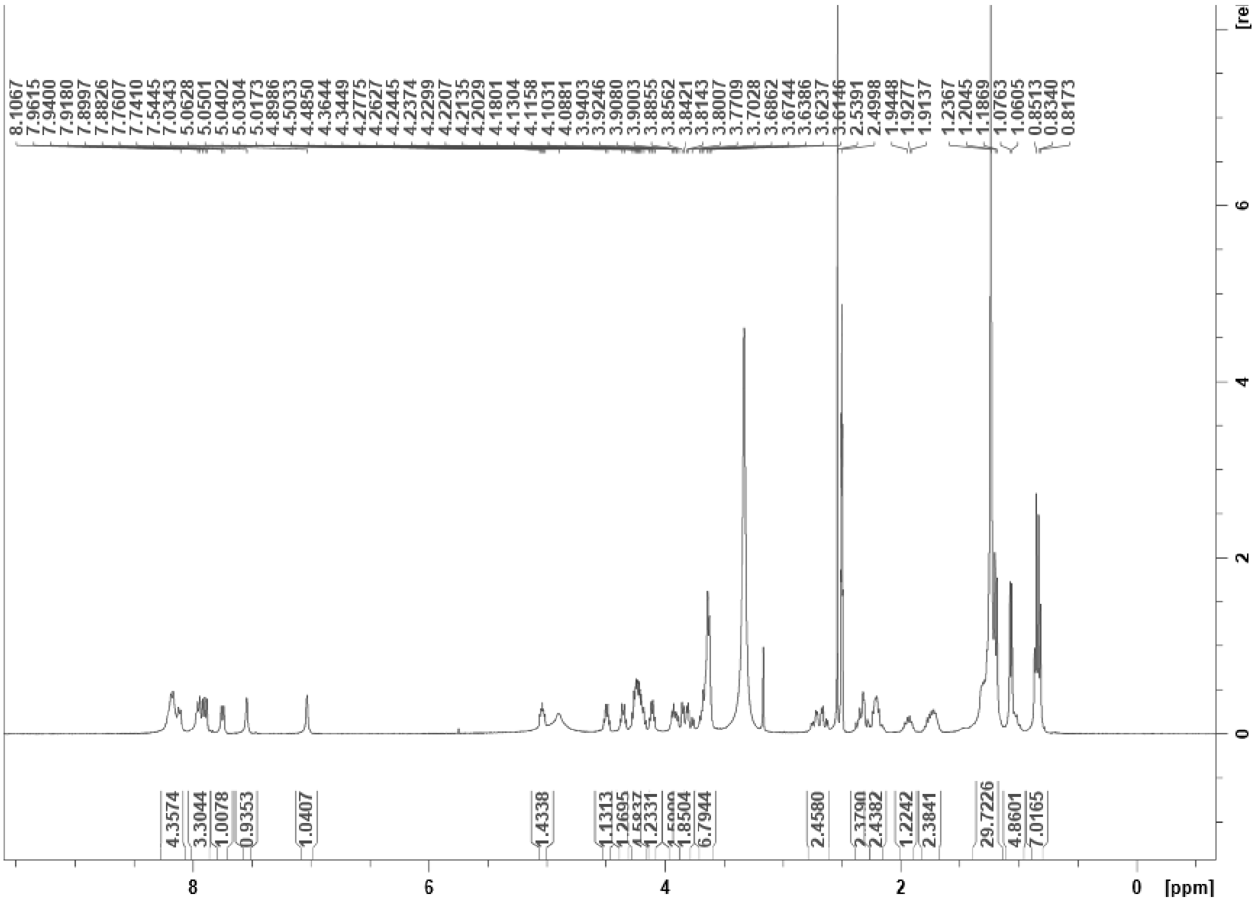

Figure S4: ^{13}C NMR spectrum (DMSO-d_6, 100 MHz) of BII-Rafflesfungin

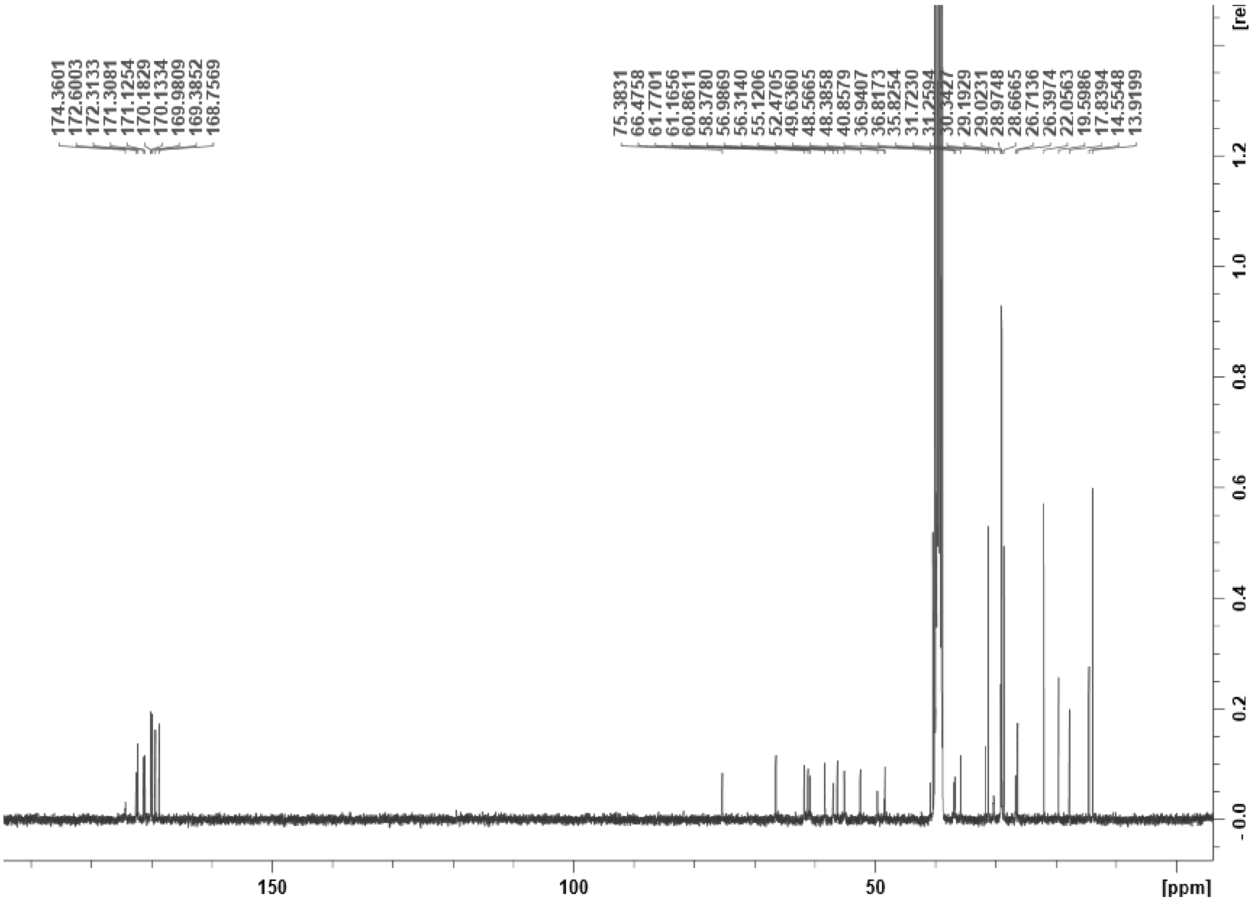

Figure S5: HSQC spectrum (DMSO-d_6, 400 MHz) of BII-Rafflesfungin

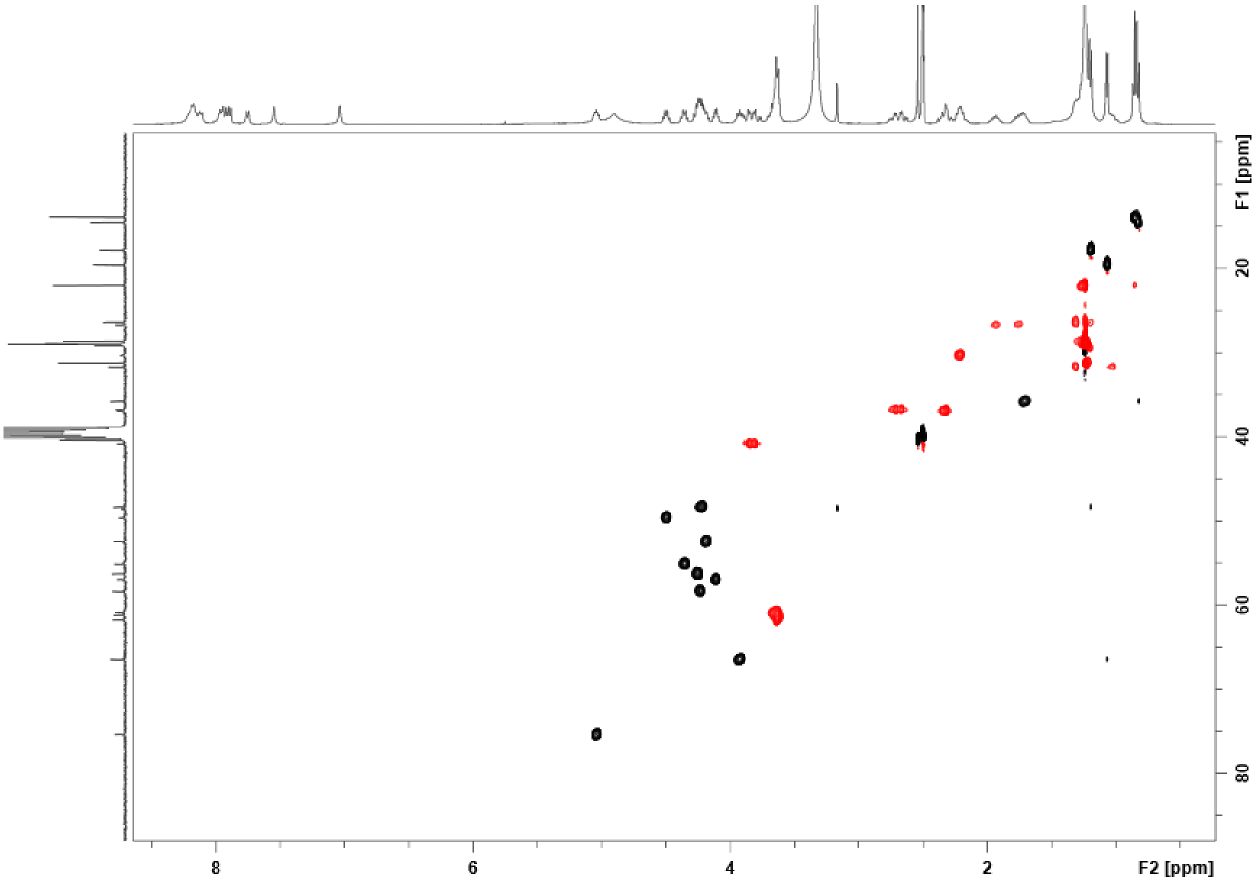

Figure S6: COSY spectrum (DMSO-d_6, 400 MHz) of BII-Rafflesfungin

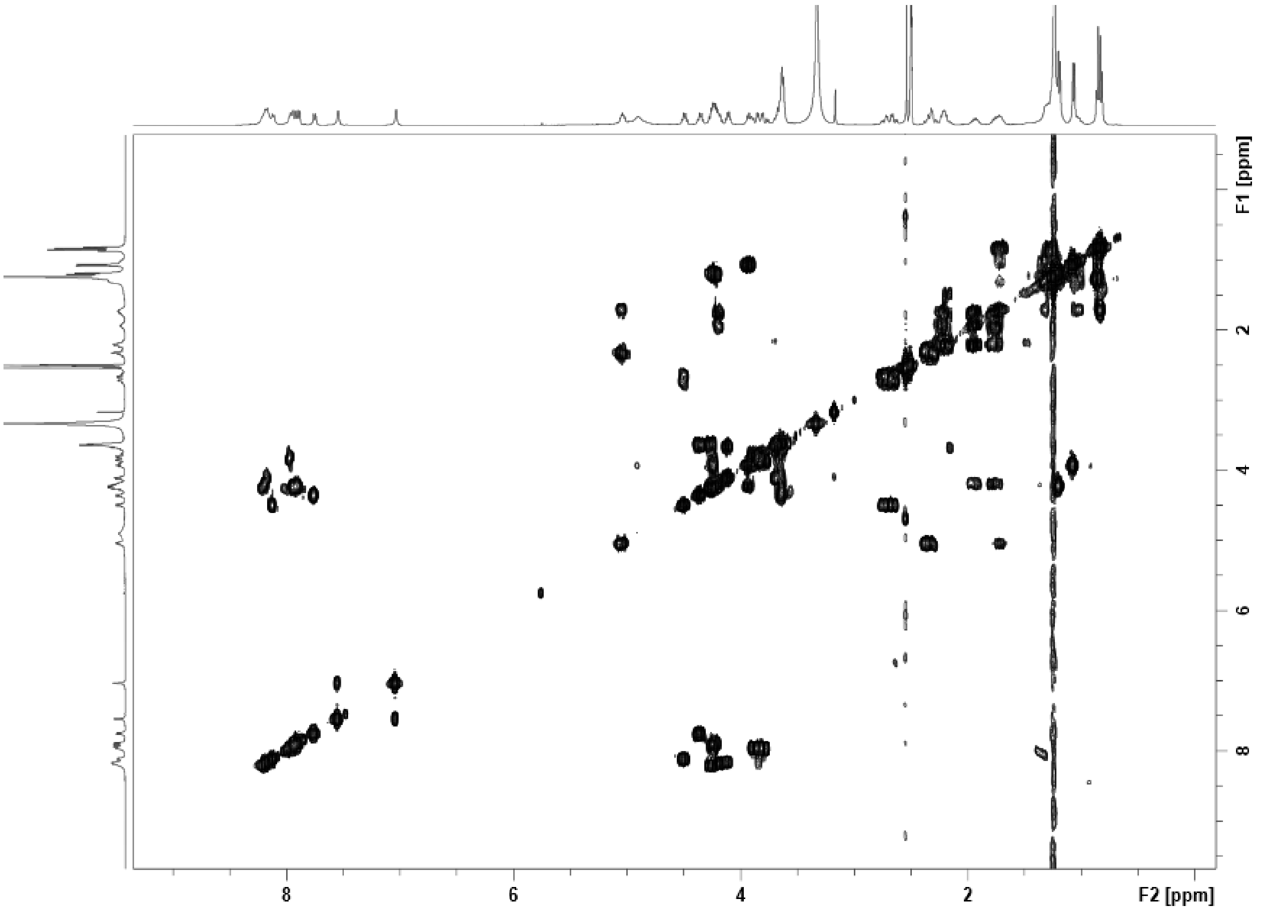

Figure S7: HMBC spectrum (DMSO-d_6, 400 MHz) of BII-Rafflesfungin

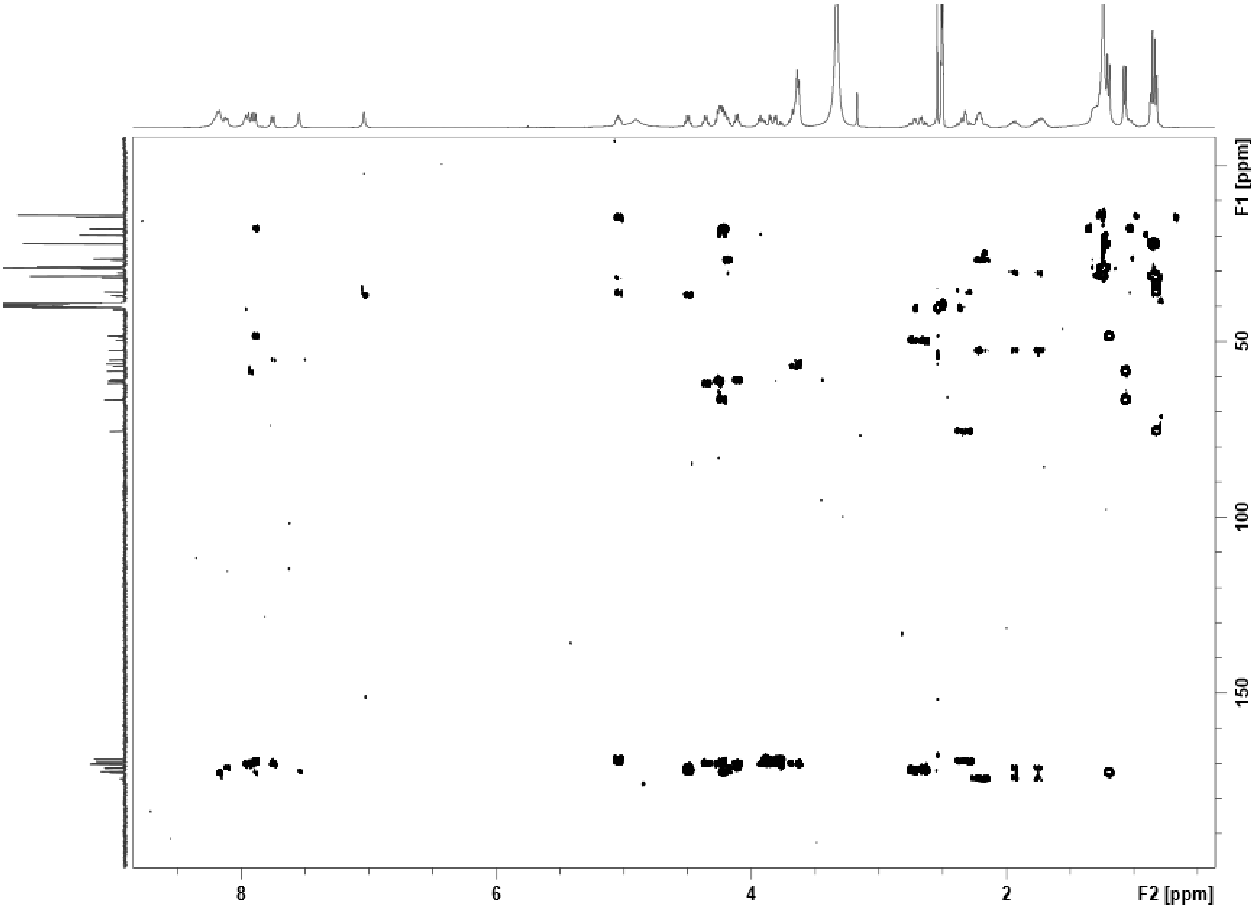

Supplement: Supplementary file 4 — Figure S3. 1H NMR spectrum (DMSO-d6, 400 MHz) of BII-Rafflesfungin. Figure S4. 13C NMR spectrum (DMSO-d6, 100 MHz) of BII-Rafflesfungin. Figure S5. HSQC spectrum (DMSO-d6, 400 MHz) of BII-Rafflesfungin. Figure S6. COSY spectrum (DMSO-d6, 400 MHz) of BII-Rafflesfungin. Figure S7. HMBC spectrum (DMSO-d6, 400 MHz) of BII-Rafflesfungin. (PDF 196 kb) [file 12864_2019_5762_MOESM4_ESM.pdf]
