# Supplementary material for: Genomics-driven discovery of a biosynthetic gene cluster required for the synthesis of BII-Rafflesfungin from the fungus Phoma sp. F3723
Source: BMC Genomics. 2019 May 14;20:374. doi: 10.1186/s12864-019-5762-6 (PMC6518819; doi:10.1186/s12864-019-5762-6)
Supplement: Supplementary file 5 — Table S2. HMMER search results against the HMM models of DCL, LCL, starter and dual type of C domains with all 9 C domains as query sequences. (PDF 78 kb) [file 12864_2019_5762_MOESM5_ESM.pdf]

Supplementary Table S2

The table shows that all the condensation domains from the *BIIRfg* cluster are highly scored by the $^{D}C_{L}$ type of C domains as compared to the other functional subtypes. The alignments for creating the HMM profiles are provided by Rausch *et. al* [1].

C Domain	$^{D}C_{L}$ HMM		$^{L}C_{L}$ HMM		Starter HMM		Dual HMM	
	Evalue	Score	Evalue	Score	Evalue	Score	Evalue	Score
C_1	**2.1e-29**	**91.6**	5.8e-27	83.7	3e-11	32.0	6.5e-18	53.9
C_2	**1.4e-40**	**128.4**	4.6e-17	51.1	1.3e-12	36.5	6.3e-19	57.2
C_3	**5.2e-39**	**123.2**	7e-18	53.7	1.7e-11	32.8	2.1e-16	48.9
C_4	**3.4e-41**	**130.4**	1.2e-15	46.3	2.1e-13	39.1	3.7e-20	61.3
C_5	**9.2e-41**	**129.0**	1.7e-17	52.5	2.1e-10	29.2	1.6e-17	52.6
C_6	**1.8e-39**	**124.7**	7.9e-15	43.7	9.4e-11	30.3	2e-16	49.0
C_7	**7.1e-47**	**149.1**	7.1e-29	90.0	2.1e-14	42.3	3e-24	74.7
C_8	**2.3e-43**	**137.6**	5.7e-25	77.1	1.5e-13	39.5	3.2e-23	71.3
C_T	**2.9e-24**	**74.7**	1.6e-13	39.4	4.1e-08	21.6	1.2e-07	20.0

Reference

1. Rausch C, Hoof I, Weber T, Wohlleben W, Huson DH: **Phylogenetic analysis of condensation domains in NRPS sheds light on their functional evolution.** *BMC Evol Biol* 2007, **7:**78.
