# Supplementary material for: Genomics-driven discovery of a biosynthetic gene cluster required for the synthesis of BII-Rafflesfungin from the fungus Phoma sp. F3723
Source: BMC Genomics. 2019 May 14;20:374. doi: 10.1186/s12864-019-5762-6 (PMC6518819; doi:10.1186/s12864-019-5762-6)
Supplement: Supplementary file 6 — Figure S8. Phylogenetic tree analysis of the functional distribution of C domains using MEGA7 (Maximum Likelihood (ML) tree). (PDF 23 kb) [file 12864_2019_5762_MOESM6_ESM.pdf]

Figure S8: Phylogenetic tree analysis of the functional distribution of C domains using MEGA7 (Maximum Likelihood (ML) tree).

The evolutionary history was inferred by using the Maximum Likelihood method based on the JTT matrix-based model [1]. The tree with the highest log likelihood (-25192.8059) is shown. The percentage of trees in which the associated taxa clustered together is shown next to the branches. Initial tree(s) for the heuristic search were obtained automatically by applying Neighbor-Join and BioNJ algorithms to a matrix of pairwise distances estimated using a JTT model, and then selecting the topology with superior log likelihood value. A discrete Gamma distribution was used to model evolutionary rate differences among sites (4 categories (+G, parameter = 1.0811)). The tree is drawn to scale, with branch lengths measured in the number of substitutions per site. The analysis involved 198 amino acid sequences. All positions containing gaps and missing data were eliminated. There were a total of 94 positions in the final dataset. Evolutionary analyses were conducted in MEGA7 [2].

References

1. Jones D.T., Taylor W.R., and Thornton J.M. (1992). The rapid generation of mutation data matrices from protein sequences. Computer Applications in the Biosciences 8: 275-282.

2. Kumar S., Stecher G., and Tamura K. (2015). MEGA7: Molecular Evolutionary Genetics Analysis version 7.0 for bigger datasets. Molecular Biology and Evolution. Mol Biol Evol. 2016 Jul;33(7):1870-4.

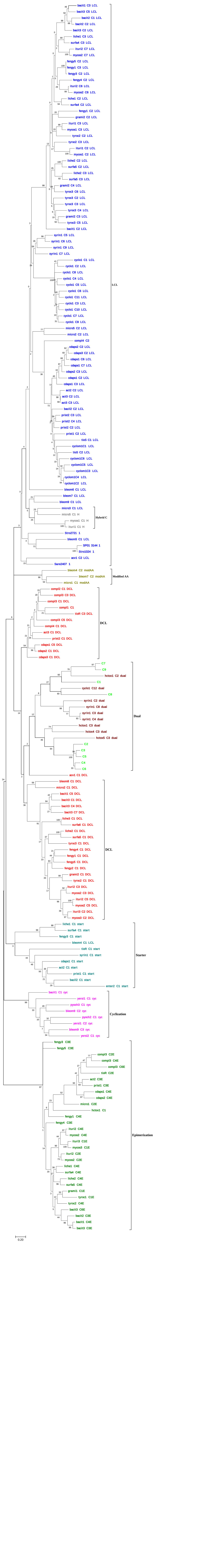
LCL
Hybrid C
Modified AA
DCL
Dual
DCL
Starter
Cyclization
Epimerization
0.20
