# Supplementary material for: Genomics-driven discovery of a biosynthetic gene cluster required for the synthesis of BII-Rafflesfungin from the fungus Phoma sp. F3723
Source: BMC Genomics. 2019 May 14;20:374. doi: 10.1186/s12864-019-5762-6 (PMC6518819; doi:10.1186/s12864-019-5762-6)

Figure S9: Multiple sequence alignment of the two epimerization domains E1 and E2 from the *BIIRfg_NRPS* gene. The motif **HHxxxDxVSW** is boxed and coloured in red.

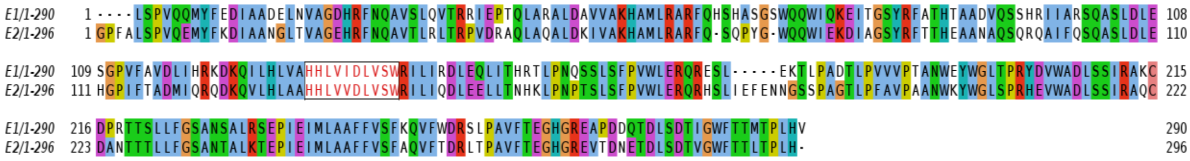

Supplement: Supplementary file 7 — Figure S9. Multiple sequence alignment of the two epimerization domains E1 and E2 from the BIIRfg_NRPS gene. (PDF 124 kb) [file 12864_2019_5762_MOESM7_ESM.pdf]
