# Supplementary material for: Genomics-driven discovery of a biosynthetic gene cluster required for the synthesis of BII-Rafflesfungin from the fungus Phoma sp. F3723
Source: BMC Genomics. 2019 May 14;20:374. doi: 10.1186/s12864-019-5762-6 (PMC6518819; doi:10.1186/s12864-019-5762-6)

Figure S10: **Results of the LCMS analysis of the absolute configuration of BII-Rafflesfungin by Marfey's method.** Extracted ion chromatograms (EIC) for ±5ppm corresponding to the $[M+H]^+$ ions of derivatized amino acids, which are present in the peptide scaffold of BII-Rafflesfungin. **a)** Hydrolyzed **BII-Rafflesfungin** derivatized with FDAA reagent. **b)** Derivatized glycine (Gly) standard with FDAA reagent. **c)** Derivatized D- and L-alanine (Ala) standards with FDAA reagent. **d)** Derivatized D- and L-aspartic acid (Asp) standards with FDAA reagent. **e)** Derivatized D- and L-glutamic acid (Glu) standards with FDAA reagent. **f)** Derivatized D- and L-serine (Ser) standards with FDAA reagent. **g)** Derivatized D- and L-threonine (Thr) standards with FDAA reagent. **h)** Derivatized D- and L-allo-threonine (allo-Thr) standards with FDAA reagent.

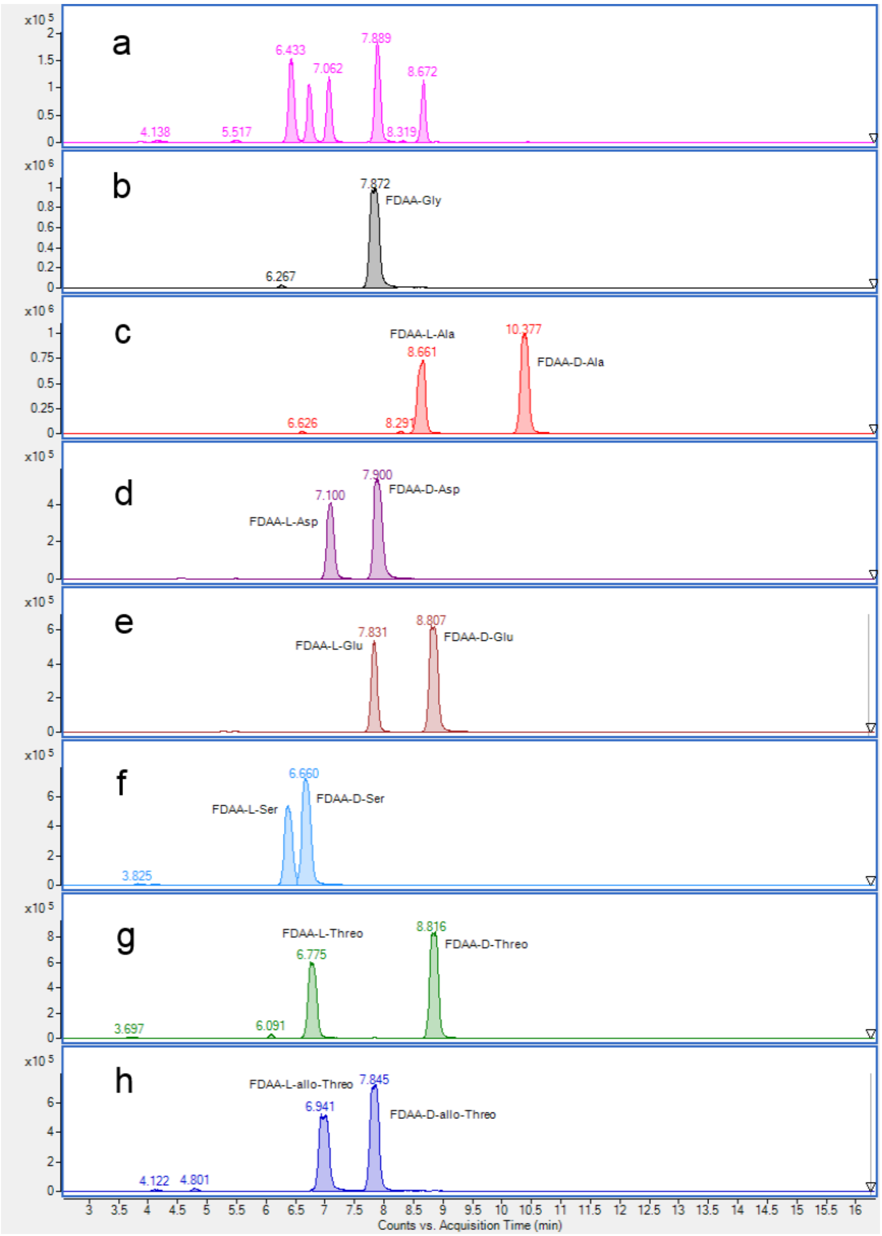

Supplement: Supplementary file 8 — Figure S10. Results of the LCMS analysis of the absolute configuration of BII-Rafflesfungin by Marfey’s method. (PDF 111 kb) [file 12864_2019_5762_MOESM8_ESM.pdf]
