# Supplementary material for: Genomics-driven discovery of a biosynthetic gene cluster required for the synthesis of BII-Rafflesfungin from the fungus Phoma sp. F3723
Source: BMC Genomics. 2019 May 14;20:374. doi: 10.1186/s12864-019-5762-6 (PMC6518819; doi:10.1186/s12864-019-5762-6)
Supplement: Supplementary file 9 — Figure S11. Multiple sequence alignment of the predicted AMP-dependent ligase for BII-Rafflesfungin biosynthesis along with VlmC (AMP-dependent ligase from Verlamelin biosynthesis) and EcdI (AMP-dependent ligase from Echinocandin B biosynthesis). (PDF 353 kb) [file 12864_2019_5762_MOESM9_ESM.pdf]

Supplementary Figure S11: Multiple sequence alignment of the predicted AMP-dependent ligase for BII-Rafflesfungin biosynthesis along with VlmC (AMP-dependent ligase from Verlamelin biosynthesis [1]) and EcdI (AMP-dependent ligase from Echinocandin B biosynthesis [2])

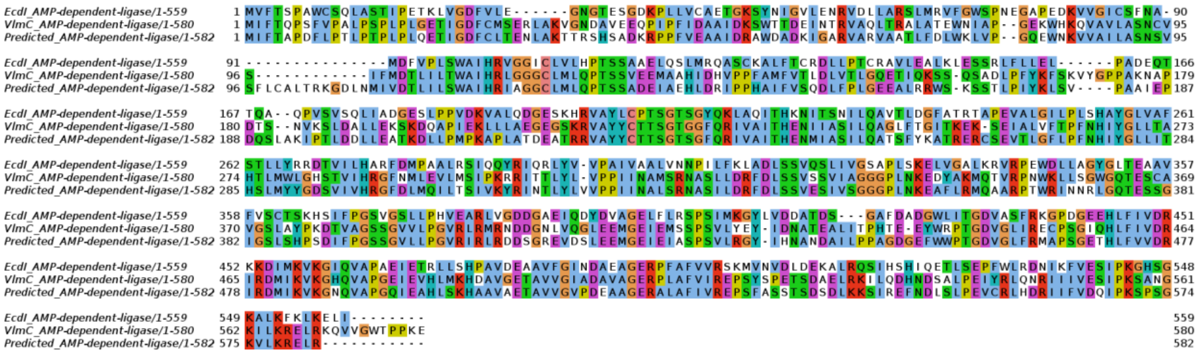

Reference

1. Ishidoh K, Kinoshita H, Nihira T: **Identification of a gene cluster responsible for the biosynthesis of cyclic lipopeptide verlamelin.** *Appl Microbiol Biotechnol* 2014, **98:**7501-7510.

2. Cacho RA, Jiang W, Chooi YH, Walsh CT, Tang Y: **Identification and characterization of the echinocandin B biosynthetic gene cluster from Emericella rugulosa NRRL 11440.** *J Am Chem Soc* 2012, **134:**16781-16790.
