# Supplementary material for: Genomics-driven discovery of a biosynthetic gene cluster required for the synthesis of BII-Rafflesfungin from the fungus Phoma sp. F3723
Source: BMC Genomics. 2019 May 14;20:374. doi: 10.1186/s12864-019-5762-6 (PMC6518819; doi:10.1186/s12864-019-5762-6)
Supplement: Supplementary file 10 — Figure S12. Multiple sequence alignment of the predicted Type II thioesterase (orf-a) along with the known TEII of Surfactin-Synthetase (SrfAD) and Rifamycin (RifR). (PDF 215 kb) [file 12864_2019_5762_MOESM10_ESM.pdf]

Supplementary Figure S12: Multiple sequence alignment of the predicted Type II thioesterase (orf-a) along with the known TEII of Surfactin-Synthetase (SrfAD) and Rifamycin (RifR).

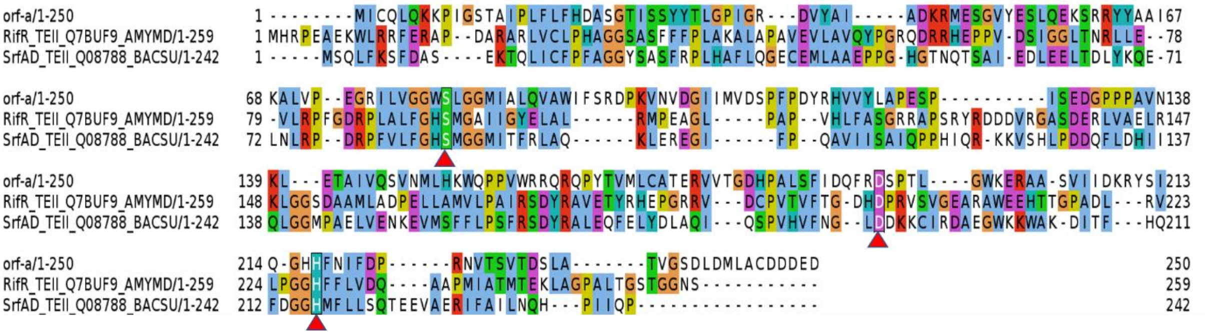

The alignment highlights the conserved active site triad using red triangles in all the three sequences, S82-D192-H217 in orf-a; S94-D200-H228 in RifR [1]; S86-D189-H216 in SrfAD [2]. The predicted orf-a protein harbours a Pfam thioesterase domain (PF00975, HMMER hit, positions 15-236 with E-value=4.2e-7) which belongs to the alpha-beta hydrolase clan (CL0028). The same sequence region also gives a structural HHPred hit to 2K2Q_B (Surfactin synthetase thioesterase subunit) with an E-value of 4.8e-24 as well as to 3FLA_A (RifR alpha-beta hydrolase thioesterase) with an Evalue of 2.7e-17.

Reference List

1. Claxton HB, Akey DL, Silver MK, Admiraal SJ, Smith JL: **Structure and functional analysis of RifR, the type II thioesterase from the rifamycin biosynthetic pathway.** *J Biol Chem* 2009, **284:**5021-5029.

2. Koglin A, Lohr F, Bernhard F, Rogov VV, Frueh DP, Strieter ER, Mofid MR, Guntert P, Wagner G, Walsh CT et al.: **Structural basis for the selectivity of the external thioesterase of the surfactin synthetase.** *Nature* 2008, **454:**907-911.
