# Supplementary material for: Genomics-driven discovery of a biosynthetic gene cluster required for the synthesis of BII-Rafflesfungin from the fungus Phoma sp. F3723
Source: BMC Genomics. 2019 May 14;20:374. doi: 10.1186/s12864-019-5762-6 (PMC6518819; doi:10.1186/s12864-019-5762-6)

Figure S13: BII-Rafflesfungin inhibits the growth of yeast cells/standard CLSI test with Amphotericin B as positive control. *C. albicans* (SC5314) cells were incubated with indicated concentrations of BII Rafflesfungin and Amphotericin B in RPMI 1640 medium in duplicates in a 96-well microplate. A picture of the microplate after 48 hours of incubation at 35°C is shown. Inhibitory Concentration (IC) of the compounds was determined as the lowest compound concentration showing no visual growth (optically clear well). IC was 32 µM for BII Rafflesfungin and 125 nM for Amphotericin B against *C. albic*ans.

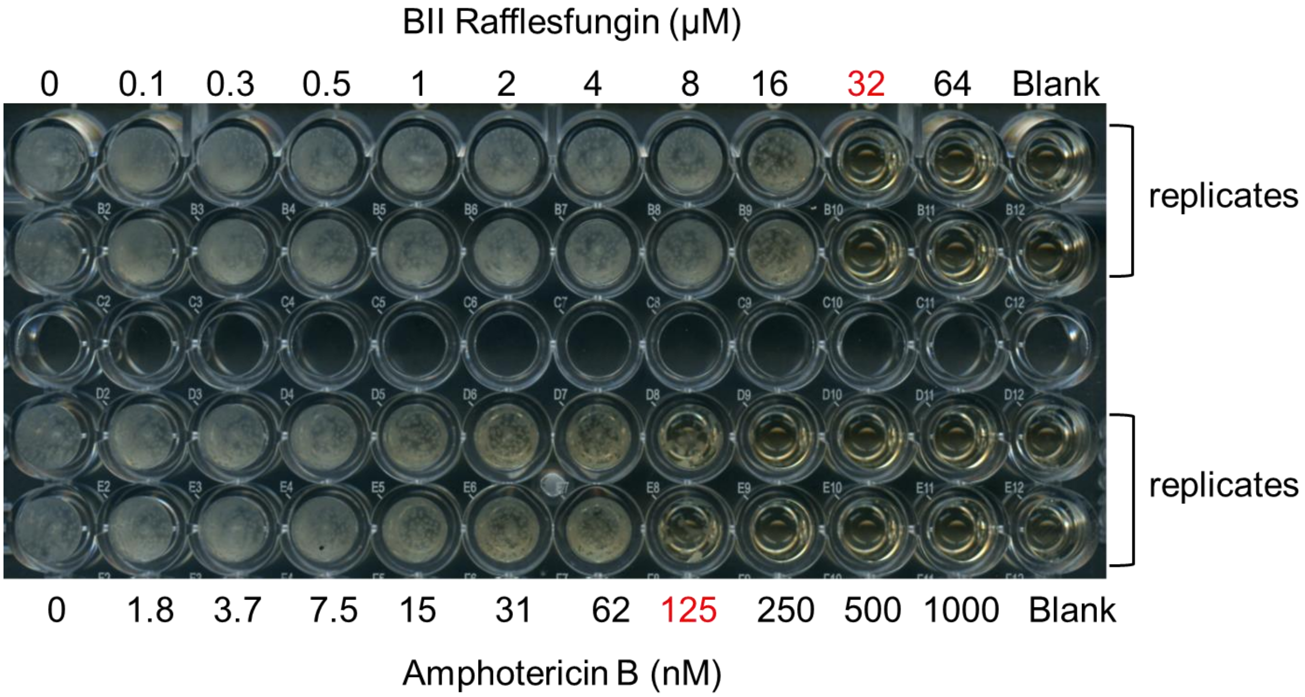

Supplement: Supplementary file 11 — Figure S13. BII-Rafflesfungin inhibits the growth of yeast cells/standard CLSI test with Amphotericin B as positive control. (PDF 224 kb) [file 12864_2019_5762_MOESM11_ESM.pdf]
