# Supplementary material for: Genomics-driven discovery of a biosynthetic gene cluster required for the synthesis of BII-Rafflesfungin from the fungus Phoma sp. F3723
Source: BMC Genomics. 2019 May 14;20:374. doi: 10.1186/s12864-019-5762-6 (PMC6518819; doi:10.1186/s12864-019-5762-6)

Supplementary Figure S14: Growth inhibitory effects of BII Rafflesfungin against yeasts (a), Aspergillus species (b) and mammalian cell lines (c).

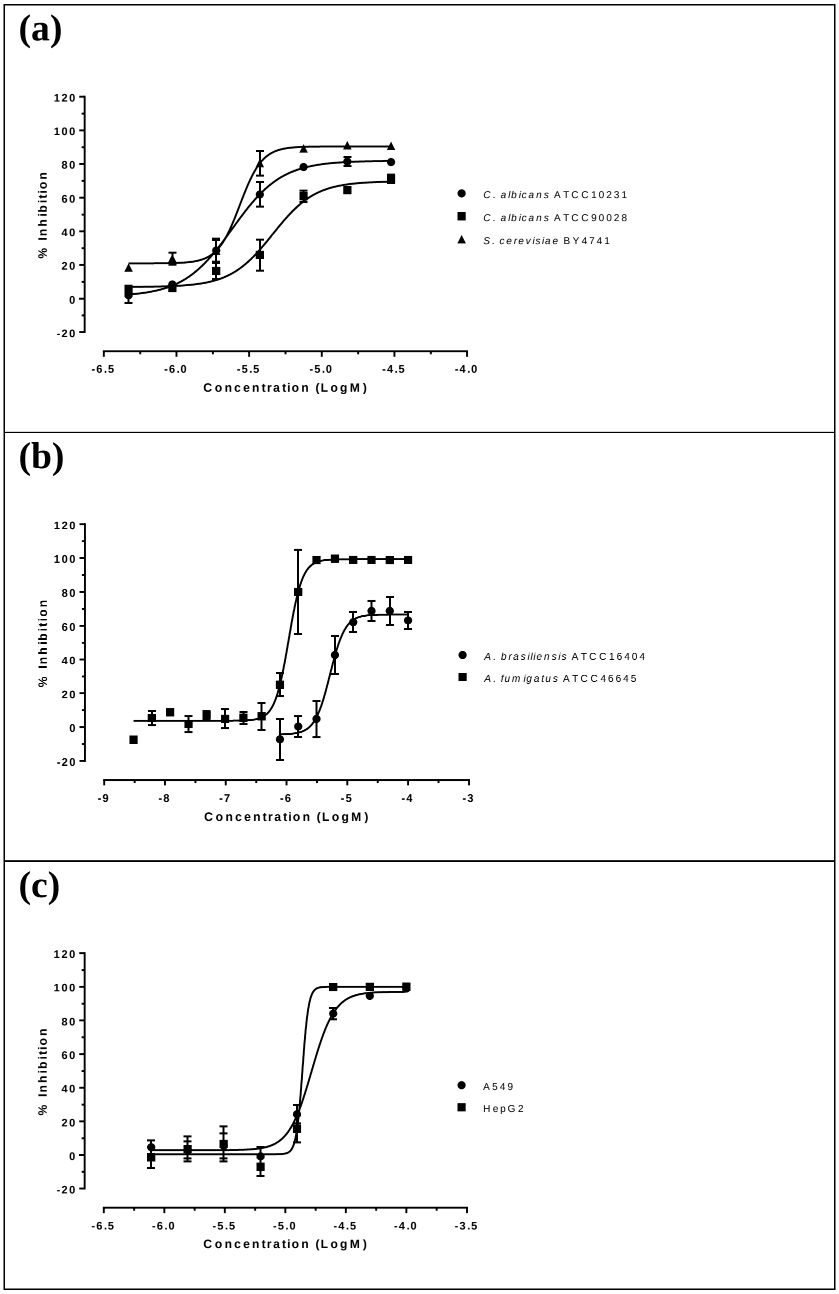

Supplement: Supplementary file 12 — Figure S14. Growth inhibitory effects of BII Rafflesfungin against yeasts (a), Aspergillus species (b) and mammalian cell lines (c). (PDF 100 kb) [file 12864_2019_5762_MOESM12_ESM.pdf]
