# Supplementary material for: Genomics-driven discovery of a biosynthetic gene cluster required for the synthesis of BII-Rafflesfungin from the fungus Phoma sp. F3723
Source: BMC Genomics. 2019 May 14;20:374. doi: 10.1186/s12864-019-5762-6 (PMC6518819; doi:10.1186/s12864-019-5762-6)
Supplement: Supplementary file 13 — Figure S15. BII-Rafflesfungin has cytocidal activity. (PDF 40 kb) [file 12864_2019_5762_MOESM13_ESM.pdf]

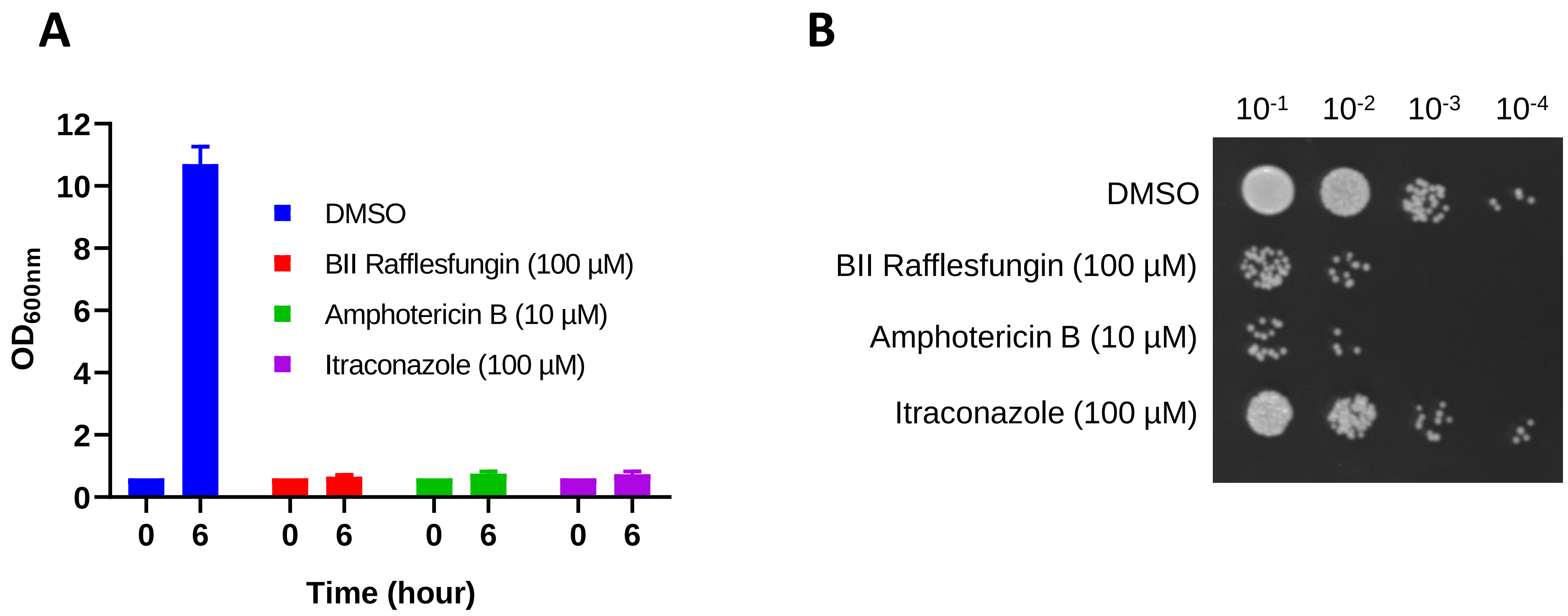


**Figure S15 BII-Rafflesfungin has cytocidal activity**

*A.* *C. albicans* (SC5314) cells at a starting $OD_{600\ nm}$ of 0.6 were incubated with either DMSO or BII-Rafflesfungin (100 µM) or Amphotericin B (10 µM) or Itraconazole (100 µM) in duplicate in YPD medium for 6 h at 30 $^{o}$C with shaking ( 220 rpm). $OD_{600\ nm}$ values of the cultures at the start and after 6 h are presented graphically.

B. Cells treated in A were washed twice, normalized and diluted in YPD medium. Equal volumes (3 µl) were spotted on YPD agar plate. Growth was recorded after incubation of the plates at 30 $^{o}$C for 2 days.
